# High-resolution microbiome profiling uncovers *Fusobacterium nucleatum*, *Lactobacillus gasseri/johnsonii*, and *Lactobacillus vaginalis* associated to oral and oropharyngeal cancer in saliva from HPV positive and HPV negative patients treated with surgery and chemo-radiation

**DOI:** 10.18632/oncotarget.20677

**Published:** 2017-09-07

**Authors:** Rafael Guerrero-Preston, James Robert White, Filipa Godoy-Vitorino, Arnold Rodríguez-Hilario, Kelvin Navarro, Herminio González, Christina Michailidi, Anne Jedlicka, Sierra Canapp, Jessica Bondy, Amanda Dziedzic, Barbara Mora-Lagos, Gustavo Rivera-Alvarez, Carmen Ili-Gangas, Priscilla Brebi-Mieville, William Westra, Wayne Koch, Hyunseok Kang, Luigi Marchionni, Young Kim, David Sidransky

**Affiliations:** ^1^ Department of Otolaryngology and Head and Neck Surgery, Johns Hopkins University, School of Medicine, Baltimore, Maryland, USA; ^2^ Department of Obstetrics and Gynecology, University of Puerto Rico, School of Medicine, San Juan, Puerto Rico; ^3^ Department of Computational Biology Resphera Biosciences, Baltimore, MD, USA; ^4^ Natural Sciences Department, Microbial Ecology and Genomics Lab, Inter American University of Puerto Rico, Metropolitan Campus, San Juan, Puerto Rico; ^5^ Department of Molecular Microbiology and Immunology, Johns Hopkins University, School of Public Health, Baltimore, Maryland, USA; ^6^ Laboratory of Molecular Pathology, Department of Pathological Anatomy, School of Medicine, Universidad de La Frontera, Temuco, Chile; ^7^ Center of Excellence in Translational Medicine - Scientific and Technological Bioresource Nucleus (CEMT-BIOREN), Universidad de La Frontera, Temuco, Chile; ^8^ Department of Oncology, Johns Hopkins University, School of Medicine, Baltimore, Maryland, USA; ^9^ Department of Otolaryngology, Vanderbilt University School of Medicine, Nashville, TN, USA

**Keywords:** microbiota, high-resolution microbiome profiling, oral cancer, oropharyngeal cancer, human microbiome project

## Abstract

Microbiome studies show altered microbiota in head and neck squamous cell carcinoma (HNSCC), both in terms of taxonomic composition and metabolic capacity. These studies utilized a traditional bioinformatics methodology, which allows for accurate taxonomic assignment down to the genus level, but cannot accurately resolve species level membership. We applied Resphera Insight, a high-resolution methodology for 16S rRNA taxonomic assignment that is able to provide species-level context in its assignments of 16S rRNA next generation sequencing (NGS) data.

Resphera Insight applied to saliva samples from HNSCC patients and healthy controls led to the discovery that a subset of HNSCC saliva samples is significantly enriched with commensal species from the vaginal flora, including *Lactobacillus gasseri/johnsonii* (710x higher in saliva) and *Lactobacillus vaginalis* (52x higher in saliva). These species were not observed in normal saliva from Johns Hopkins patients, nor in 16S rRNA NGS saliva samples from the Human Microbiome Project (HMP). Interestingly, both species were only observed in saliva from Human Papilloma Virus (HPV) positive and HPV negative oropharyngeal cancer patients. We confirmed the representation of both species in HMP data obtained from mid-vagina (n=128) and vaginal introitus (n=121) samples.

Resphera Insight also led to the discovery that *Fusobacterium nucleatum*, an oral cavity flora commensal bacterium linked to colon cancer, is enriched (600x higher) in saliva from a subset of HNSCC patients with advanced tumors stages.

Together, these high-resolution analyses on 583 samples suggest a possible role for bacterial species in the therapeutic outcome of HPV positive and HPV negative HNSCC patients.

## INTRODUCTION

Microorganisms cause an estimated 20% of cancer in humans [1, 2]. The best-known example is the role *Helicobater pylori* plays in gastric cancer [3–5]. A handful of laboratories have reported links between the human microbiome, immuno modulators, inflammation and tumor initiation or progression in oral [6], colon [7–10], pancreatic [7, 11], liver [12], esophageal [13] and prostate cancers [14]. The biological mechanism of these associations is not yet understood [15, 16].

Cancer initiation, development, metastasis and its response to therapy are shaped by site specific genomic, epigenomic and immunologic alterations, all of which are related to acute or chronic inflammatory states, first described by Virchow 150 years ago [17, 18]. Four different types of inflammation seem to precede cancer initiation: 1) chronic inflammation associated to infections or autoimmune disorders; 2) low grade chronic inflammation associated to environmental irritants, health behaviors, or obesity; 3) tumor associated inflammation; 4) therapy-induced inflammation [19]. Cancer initiation and progression by oncogenic mutations, genomic instability, early tumor promotion, and enhanced angiogenesis are changes linked to chronic inflammation associated to multiple etiologies [20]. Tumor-associated inflammation enhances angiogenesis, promotes tumor progression and metastatic spread, and causes local immunosuppression [19]. Therapy-induced inflammation is linked to trauma, necrosis, and tissue injury, which stimulate tumor re-emergence and resistance to therapy [21, 22]. Conversely, therapy-induced inflammation can also enhance antigen presentation, leading to immune-mediated tumor eradication [23].

The role of microbes and viruses in cancer development is also attributed to a wide spectrum of focalized changes driven by innate and adaptive immune responses [20]. There are several mechanisms by which bacterial infection can lead to the initiation and progression of oncogenic processes [2, 24, 25]. Most pathogen-induced tumors are preceded by pathogen, tissue and site specific host-mediated inflammatory states [26]. The normal tissue-repair response to injury and infection is orchestrated by an evolutionary conserved sequence of molecular changes triggered by pattern-recognition receptors (PRRs), many of which belong to the Toll-like receptor (TLR) family [27]. PPRs recognize pathogen associated molecular patterns (PAMPs) or damage associated molecular patterns (DAMPs) setting off a cascade of events that activate the innate immune response [27–30]. Bacterial endotoxins, metabolic byproducts of bacterial infection, and increased enzymatic activity as a result of bacterial infection, can induce somatic mutations and signaling pathway alterations [31].

Head neck squamous cell carcinomas (HNSCC) is a diverse group of tumor types, originally classified by anatomic subsite, but more recently best understood in terms of etiology, molecular drivers and immune phenotype [32, 33]. During the past twenty years the genomic and epigenomic changes in tumor development and treatment of HNSCC have been mapped [34–42]. These studies have revealed that the inactivation of the p53 and retinoblastoma (pRb) pathways are some of the earliest changes seen in both, HPV negative and HPV positive HNSSC patients. Inactivation of p53 and pRb in HPV negative tumors is due to the accumulation of somatic mutations and and/or promoter methylation events, while HPV positive tumors express the HPV oncoproteins E6 and E7, which also silence p53 and pRb [39, 41, 43, 44].

The method of excellence for performing initial microbiome characterization is sequencing the 16S rRNA gene to identify stable phylogenetic markers of taxonomic lineages for archaea and bacteria in a given sample [45, 46]. The 16S rRNA gene has nine hypervariable regions, including a combination of variable and moderately conserved regions optimal for performing analyses at different phylogenetic depths. The V3-V5 region of the 16S rRNA gene is one of the preferred regions for characterizing the communities with few errors in taxonomic assignment [47–49].

Recently, we analyzed 16S rRNA amplicon sequencing data using a standard bioinformatics pipeline to unveil novel characteristics of the saliva microbiome from head and neck cancer patients treated for oral cancer, as well as for HPV positive and HPV negative oropharyngeal cancer [20]. Longitudinal analyses of samples taken before and after surgery, revealed a reduction in the alpha diversity measure after surgery, together with an increase of this measure in patients that recurred (p<0.05). HNSCC patients had a significant loss in richness and diversity of microbiota species (p<0.05) compared to the controls. Overall, network analysis of operational taxonomic units (OTUs) demonstrated that the relative abundance of OTUs within genus *Streptococcus, Dialister*, and *Veillonella* could be used to discriminate HNSCC from control samples (p<0.05).

In this manuscript, we report the results of a novel analysis of this 16S rRNA dataset that allowed us to identify new associations between species in the saliva microbiome and tumor characteristics in the same HNSCC patient population treated in Johns Hopkins School of Medicine (JHU). We applied the Resphera Insight tool for high-resolution taxonomic assignment [50, 51], to perform a cross-sectional comparison at the species level of the microbial communities present in saliva DNA from HPV positive and HPV negative patients with cancer of the oropharynx, cancer of the oral cavity, and participants with normal oral cavity epithelium. We also selected between 1-4 additional saliva samples collected in subsequent visits, from 10 of the 19 HNSCC patients to evaluate the longitudinal association between microbial species abundance and community members and treatment effects. We then confirmed the results obtained on the saliva samples from the JHU Discovery cohort on 514 samples from the Human Microbiome Project (HMP) [52].

## RESULTS

**Figure 1:**
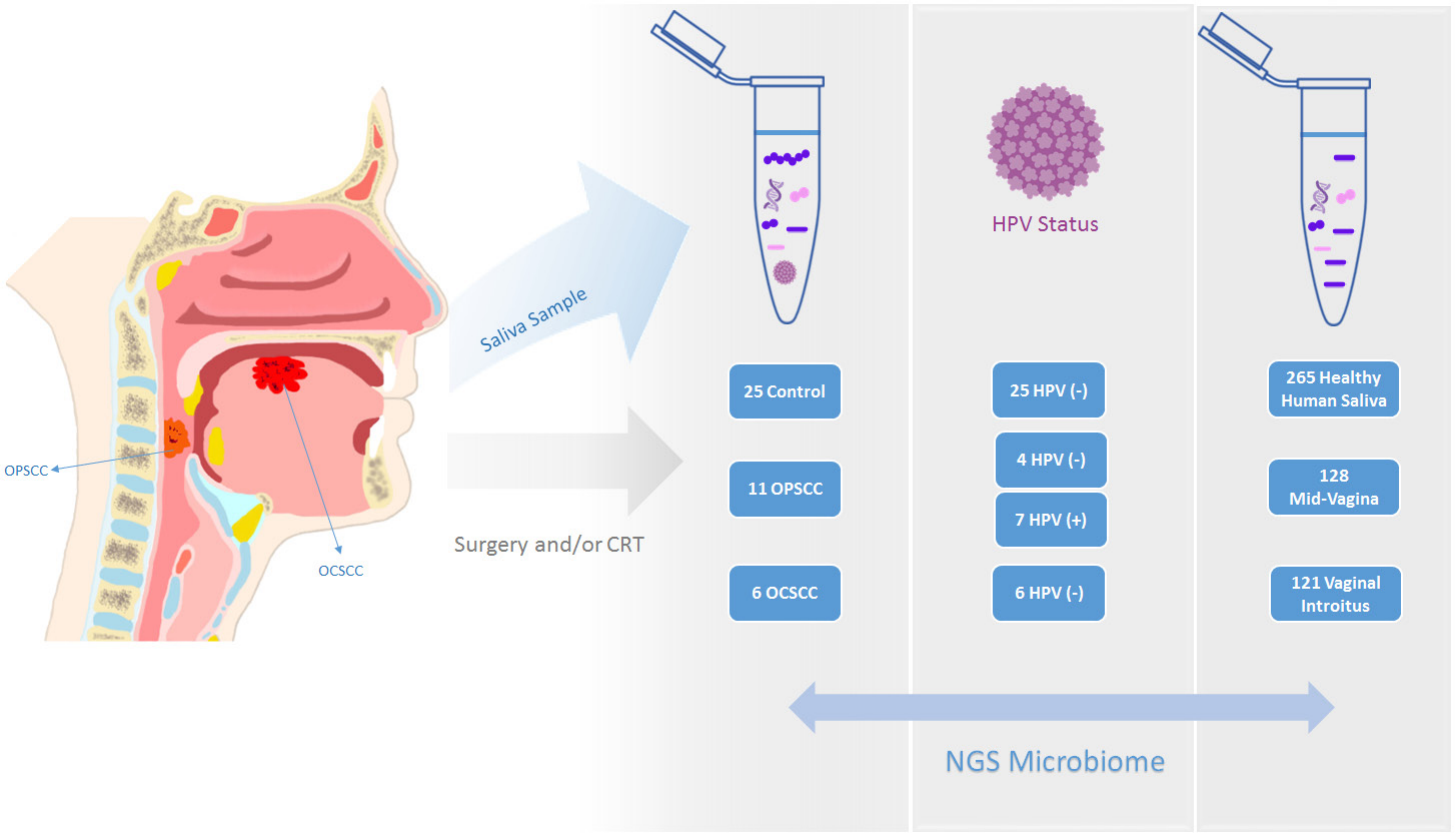
We utilized a quality-controlled set of clean sequences obtained by sequencing in a Roche-454 FLX the 16S rRNA V3V5 region from 59 saliva DNA samples (17 HNSCC patients and 25 normal controls; JHU Cohort) Additionally, we obtained high-quality 16S rRNA sequence datasets obtained with V3V5 region / Roche-454 FLX sequencing on 514 samples from 154 unique participants in the Human Microbiome Project.

Microbiome studies have revealed altered taxonomic composition in HNSCC saliva and tissue samples using a traditional bioinformatics methodology, which allows for accurate taxonomic assignment down to the genus level, but cannot accurately resolve species level membership. We applied Resphera Insight to classify bacterial species present in a quality-controlled set of clean 16S rRNA sequences previously analyzed by our group [20]. We first classified the species present in sequence datasets obtained with V3V5 region / Roche-454 FLX sequencing on 59 saliva DNA samples (17 HNSCC patients and 25 normal controls; JHU Cohort). We then classified the species present in high-quality 16S rRNA sequence datasets obtained with V3V5 region / Roche-454 FLX sequencing on 514 samples from 154 unique participants in the Human Microbiome Project (Supplementary Tables 1a and 1b): healthy human saliva (n=265), mid-vagina (n=128), and vaginal introitus (n=121) samples (Figure 1).

High-quality 16S rRNA sequences were analyzed with Resphera Insight, a high-resolution methodology for 16S rRNA taxonomic assignment that is able to provide species-level context in its assignments (see Methods). A novel feature of the approach, when Resphera Insight is unable to make a confident single species assignment, the method will then provide an ambiguous assignment listing the specific set of species it cannot decide among. For example, an assignment of *Lactobacillus_gasseri:Lactobacillus_johnsonii* could be narrowed to within these two species, but the method could not resolve the assignment further.

We found a total of 5 assigned phyla dominating across the samples: *Firmicutes, Actinobacteria, Bacteroidetes, Fusobacteria, and Proteobacteria*. (Supplementary Figure 1A). Overall, species-level profiles showed a dominance of *Veilonella dispar* across all samples (Supplementary Figure 1B). *Streptococcus salivarius:Streptococcus vestibularis* abundances were more dominant in HNSCC samples.

Hierarchical clustering of taxonomic profiles (top 50 species level calls) shows that the salivary microbiome is distinct in HNSCC, with significant enrichment of *Lactobacillus spp, Parvimonas micra, Streptococcus mutans, and Fusobacterium nucleatum* (p < 0.05) in HNSCC samples and overall reduced alpha diversity (p < 1e-4) (Supplementary Figure 1C). We also found a larger percentage of *Fusobacterium_nucleatum* present in a subset of HNSCC saliva samples when compared with controls. Remarkably there is a highly significant depletion of *Fusobacterium periodonticum, Leptotrichia trevisanii, Leptotrichia hofstadii*, and *Leptotrichia buccalis* in HNSCC compared to controls in the JHU cohort (Supplementary Figure 2A- 2E). *Fusobacterium_nucleatum* is closely related to each of these other species. Given these unexpected findings we decided to validate our results using publicly available saliva, vaginal introitus and mid-vagina samples from the Human Microbiome Project.

**Figure 2:**
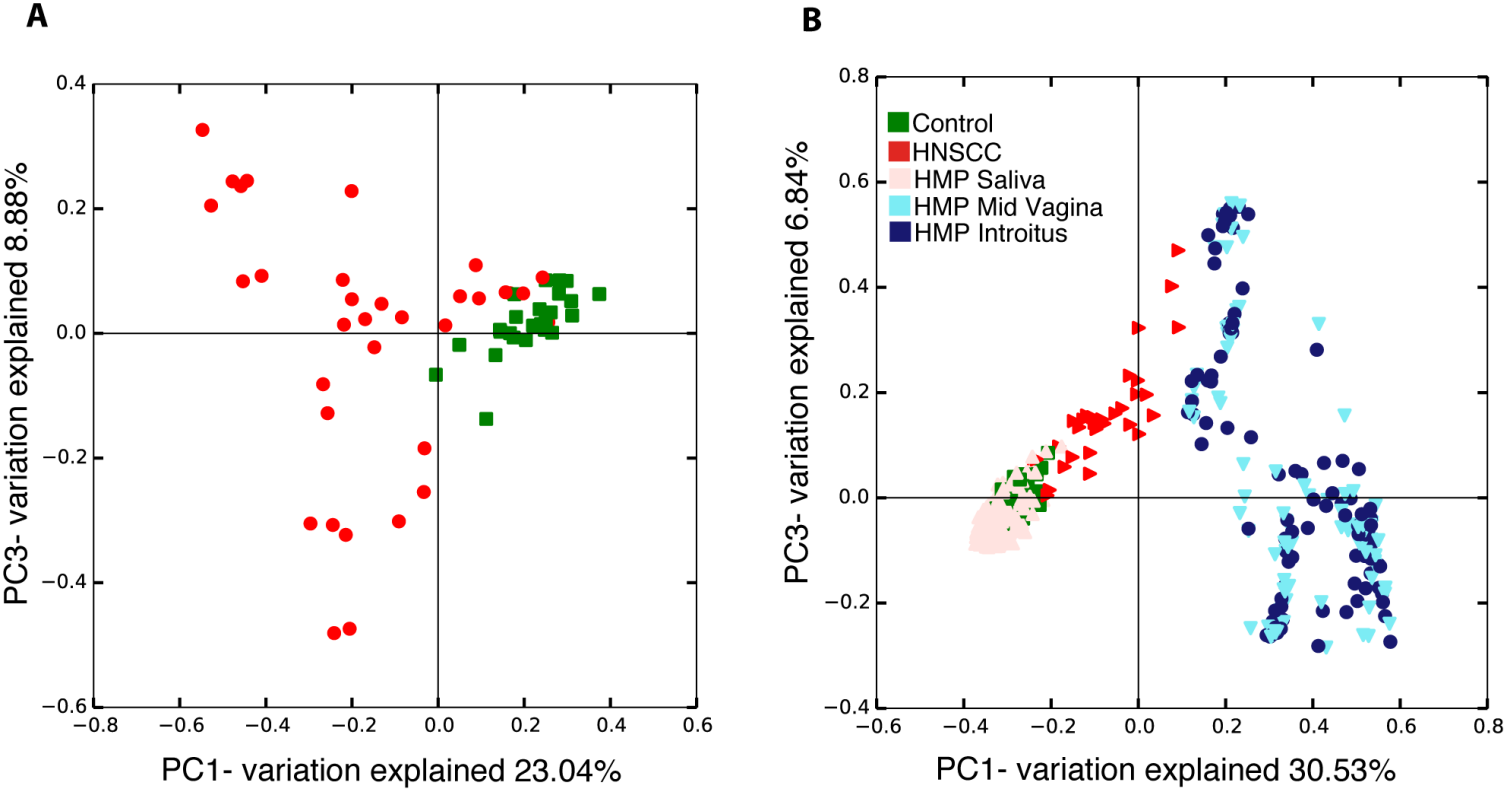
Beta diversity comparisons by Principal Component Analysis (PCoA) discriminate: **(A)** Head and neck squamous cell carcinoma (n=17) from Control samples (n=25); **(B)** All 59 JHU saliva samples from three Human Microbiome Project datasets: saliva, mid vagina and introitus.

Principal coordinates analysis (PCoA) of the Bray-Curtis distances (β-diversity) show differences in community structure in saliva samples from HNSCC when compared to normal patients (Figure 2A). Principal coordinates analysis also demonstrated differences in community structure when comparing all 59 saliva samples from JHU patients with the 514 normal samples from the HMP, as expected (Figure 2B). Surprisingly, we observed clustering of HMP and JHU samples, which was unexpected because of the extremely strong processing signal present in microbiome samples. We found dense clustering between HMP saliva from normal participants and JHU control samples after performing PCoA on the combined JHU and HMP samples. Saliva samples from normal HMP and JHU cohorts clearly overlap in two of the four quadrants (Figure 2B). Similarly unexpected, a subset of JHU HNSCC samples cluster in the same quadrant as a subset of HMP mid-vagina and introitus samples, after PCoA was performed on the combined JHU and HMP samples. These results suggest that all normal and a subset of HNSCC samples from JHU, share components with samples from the HMP.

Microbial communities from control samples display significantly higher species richness (p<0.001) than HNSCC samples (Supplementary Figure 3A) regardless of whether they are HPV positive or HPV negative samples (Supplementary Figure 3B). When examining Control and HNSCC samples with HMP saliva and vaginal samples, we found that Control and HMP saliva samples had the highest richness index followed by HNSCC and HMP vaginal samples (Supplementary Figure 3C). No significant differences were observed (p=0.15) when comparing HPV positive and HPV negative HNSCC samples with JHU Control and HMP saliva and vaginal samples (Supplementary Figure 3D).

**Figure 3:**
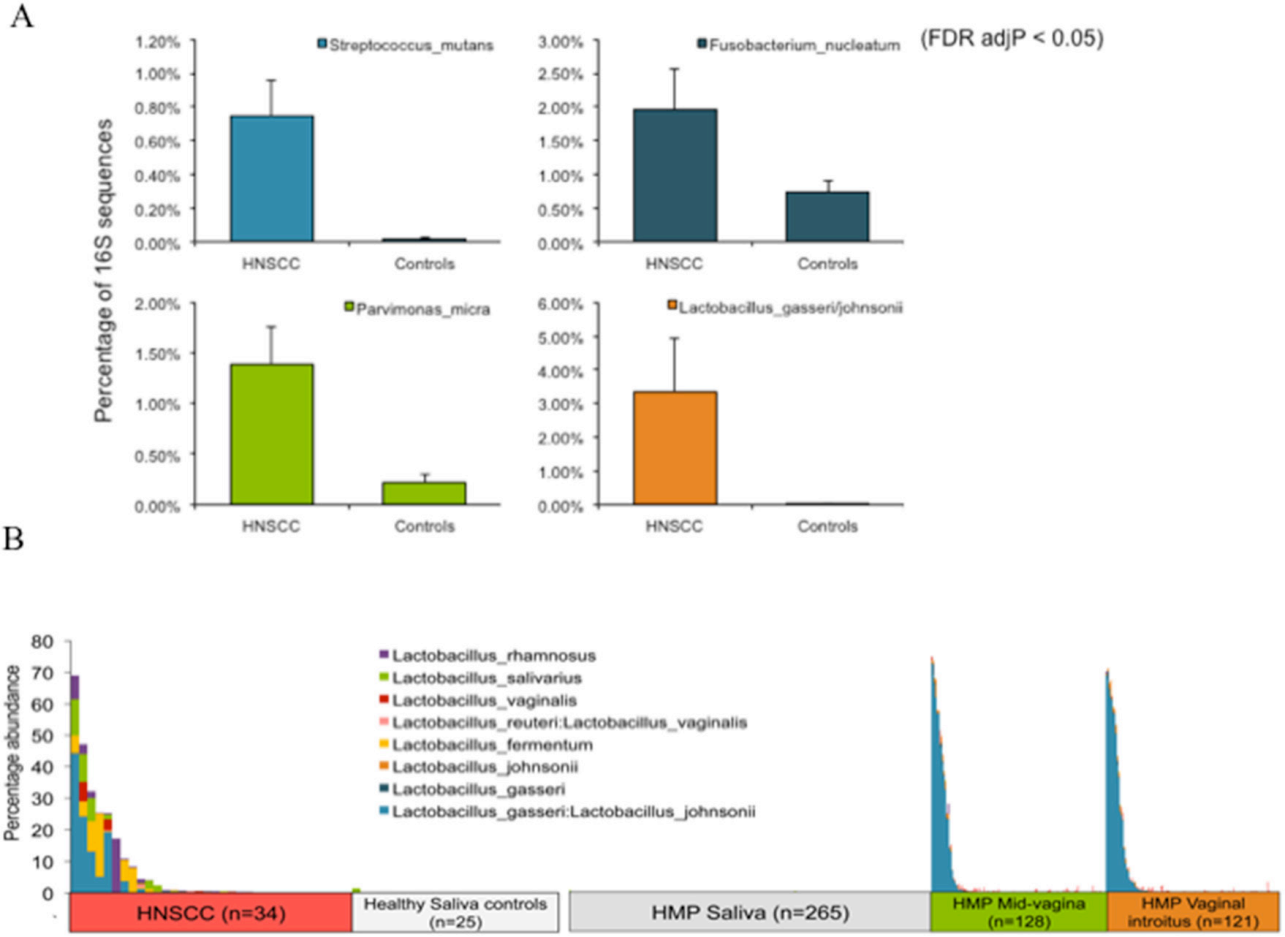
**(A)** Frequency of significant reads for Streptococcus_mutans, Fusobacterium nucleatum, Parvimonas micra and Lactobacillus gasseri:johnsonnii **(B)** Relative abundance of significant 16S rRNA NGS reads for Lactobaccillus (p<0. 05), when comparing saliva from HNSCC patients with saliva from normal patients from Johns Hopkins saliva from normal participants in the Human Microbiome Project (HMP), as well as vaginal_introitus, and mid_vagina samples from participants in the HMP.

There were no differences in the microbial phyla present in the saliva control samples from JHU and saliva from participants in the HMP observed in the area plots of taxonomic summary for phyla (Supplementary Figure 1A). Interestingly, at the species level (Supplementary Figure 1B) we found bacteria commonly seen in the vaginal flora in saliva samples from a subset of Oropharyngeal Squamous Cell Carcinoma (OPSCC) patients. A subsequent analysis of the percentage of significant reads for these cohorts revealed that *Fusobacterium nucleatum*, an oral cavity flora commensal bacterium linked to colon cancer, is enriched (600x higher) in saliva from HNSCC patients (p < 0.05) (Figure 3A). This analysis also uncovered that Lactobacillus_rhamnosus, Lactobacillus_salivarius, Lactobacillus_vaginalis; Lactobacillus_reuteri:Lactobacillus_vaginalis; Lactobacillus_fermentum, Lactobacillus_johnsoni, Lactobacillus_gasseri, and *Lactobacillus_gasseri:Lactobacillus_johnsonii* were all enriched in a subset of HNSCC saliva samples from JHU, but not in normal saliva samples from JHU or saliva samples from the HMP. Most notably, *Lactobacillus gasseri:Lactobacillus johnsonii* was 710x higher in HNSCC and *Lactobacillus vaginalis* was 52x higher in HNSCC compared to controls (p<0.05). *Lactobacillus_gasseri:Lactobacillus_johnsonii* and *Lactobacillus_gasseri* were also present in vaginal introitus and mid-vagina samples from the HMP (p < 0.05) (Figure 3B). We observed differences in abundance of significant *Lactobacillus vaginalis* and *Lactobacillus_gasseri:Lactobacillus_johnsonii* reads in a subset of HPV positive and negative OPSCC (Supplementary Figure 4A). The relative enrichment is mostly seen in patients with larger tumors and nodal involvement (T3, N2) (Supplementary Figure 4B) and only in patients treated with multimodality therapy involving surgery and chemoradiation, when compared with patients only treated with surgical removal of the tumor (Supplementary Figure 4C).

**Figure 4:**
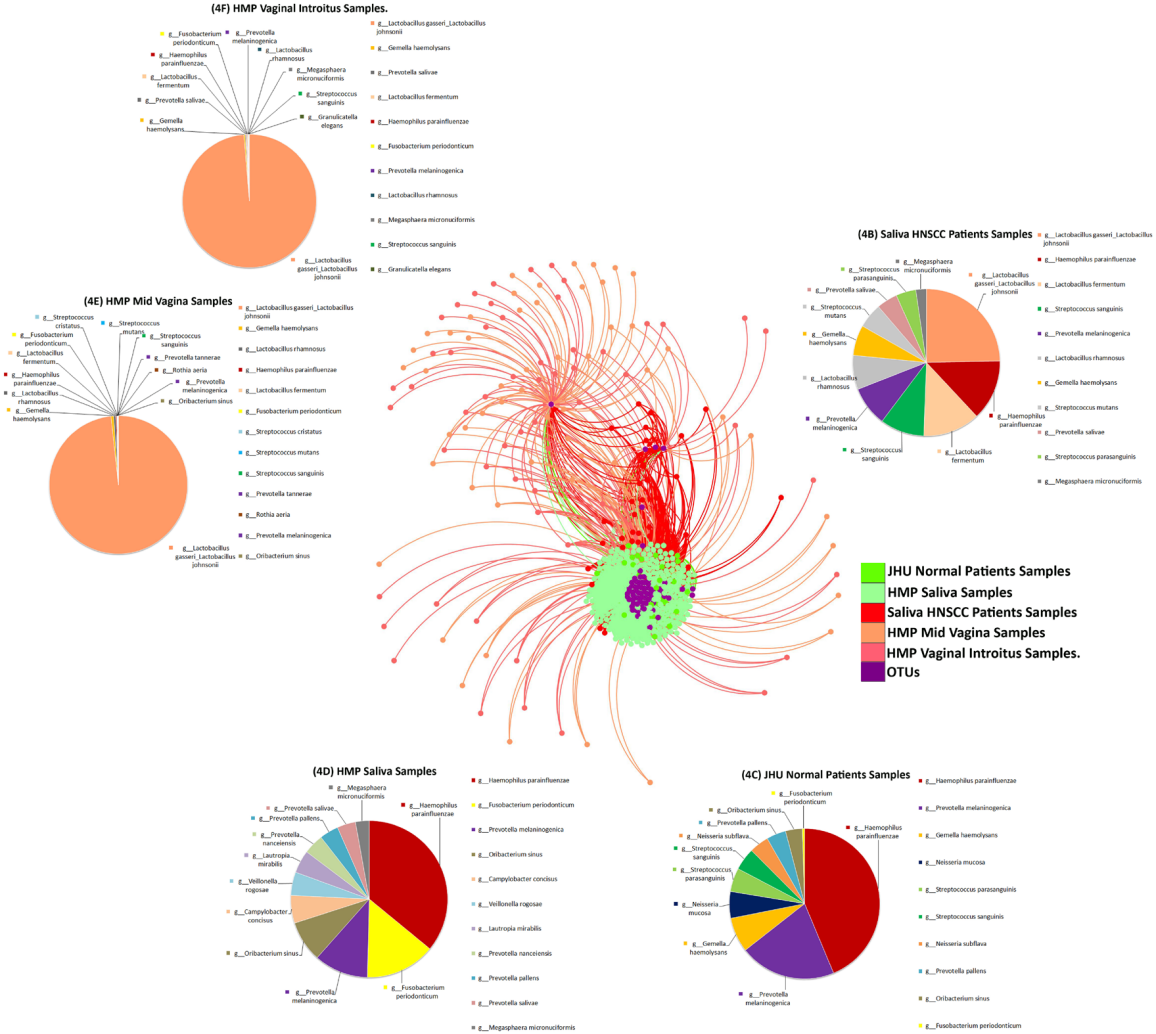
**(A)** Bacterial network of Operational Taxonomic Units at the species level that differed significantly in relative abundance (p<0. 05), comparing saliva from normal patients from Johns Hopkins saliva from normal participants in the Human Microbiome Project (HMP), as well as vaginal_introitus, and mid_vagina samples from participants in the HMP. Pie charts representing taxa that differed significantly in relative abundance (p<0.05) when comparing **(B)** saliva from HNSCC patients, **(C)** JHU normal patients, **(D)** HMP saliva samples, **(E)** HMP Mid_Vagina and **(F)** HMP Vaginal_Introitus samples.

The bacterial OTU network shown in Figure 4 represents species that differed significantly in relative abundance (p<0.05) when comparing saliva from normal patients with patients with HNSCC, as well as HPV negative and HPV positive patients. Results for this algorithm shows various clustering in according to metadata for HNSCC, JHU Control Samples, HMP Saliva Samples, HMP mid-vaginal samples and vaginal introitus samples (Figure 4A). Pie charts were created showcasing taxa distinguishing JHU samples by histology and HMP by body compartment. Pie charts represent taxa that differed significantly in relative abundance (p<0.05) when comparing saliva from HNSCC patients (Figure 4B), JHU normal patients (Figure 4C), as well as HMP saliva samples (Figure 4D), HMP mid-vagina samples (Figure 4E) and HMP vaginal samples (Figure 4F). The OTU network shows that the total abundance *Lactobacillus gasseri*/johnsonii, *Haemophilus parainfluenza, Lactobacillus fermentum and Fusobacterium periodonticum* can be used to discriminate HNSCC samples from control samples. Similarly, saliva samples from HNSCC patients, HMP mid-vaginal and vaginal introitus samples have a high abundance of *Lactobacillus gasseri*/*johnsonii*, commensal species of the vaginal flora.

We further examined the differential enrichment of bacterial OTUs in HNSCC saliva samples when compared to JHU saliva samples from normal patients. Sequencedata was log transformed and differential abundance plotted usingthe DESeq2 variance stabilization function available in Bioconductor. Single species significance tests (raw pvalue <0.05) were performed by one-way ANOVA. A single species heatmap using Euclidean distance to cluster 59 statistically significant JHU control and HNSCC samples’ OTUs (p<0.05) with the Hierarchical Unweighted Pair Group Method with Arithmetic Mean (UPGMA), shows that *Streptococcus mutants, Lactobacillus fermentum* and *Lactobacillus rhamnosus* are enriched in HNSCC samples (Supplementary Figure 5A). A single species heatmap using Euclidean distance to cluster 573 statistically significant JHU and HMP samples’ OTUs (p<0.05) with the Hierarchical Unweighted Pair Group Method with Arithmetic Mean (UPGMA), shows that *Streptococcus_salivarius: Streptococcus_vestibularis*, *Fusobacterium nucleatum*, *Prevotella oris*, *Rothia_mucilaginosa*, and *Lactobacilus_gasseri:Lactobacillus_johnsonii* are enriched in HNSCC samples, when compared to normal saliva samples from JHU and HMP. We also observed a loss of enrichment of *Prevotella_jejuni:Prevotella melaninogenica*, and *Prevotella_pallens* in HNSCC (Supplementary Figure 5B).

**Figure 5:**
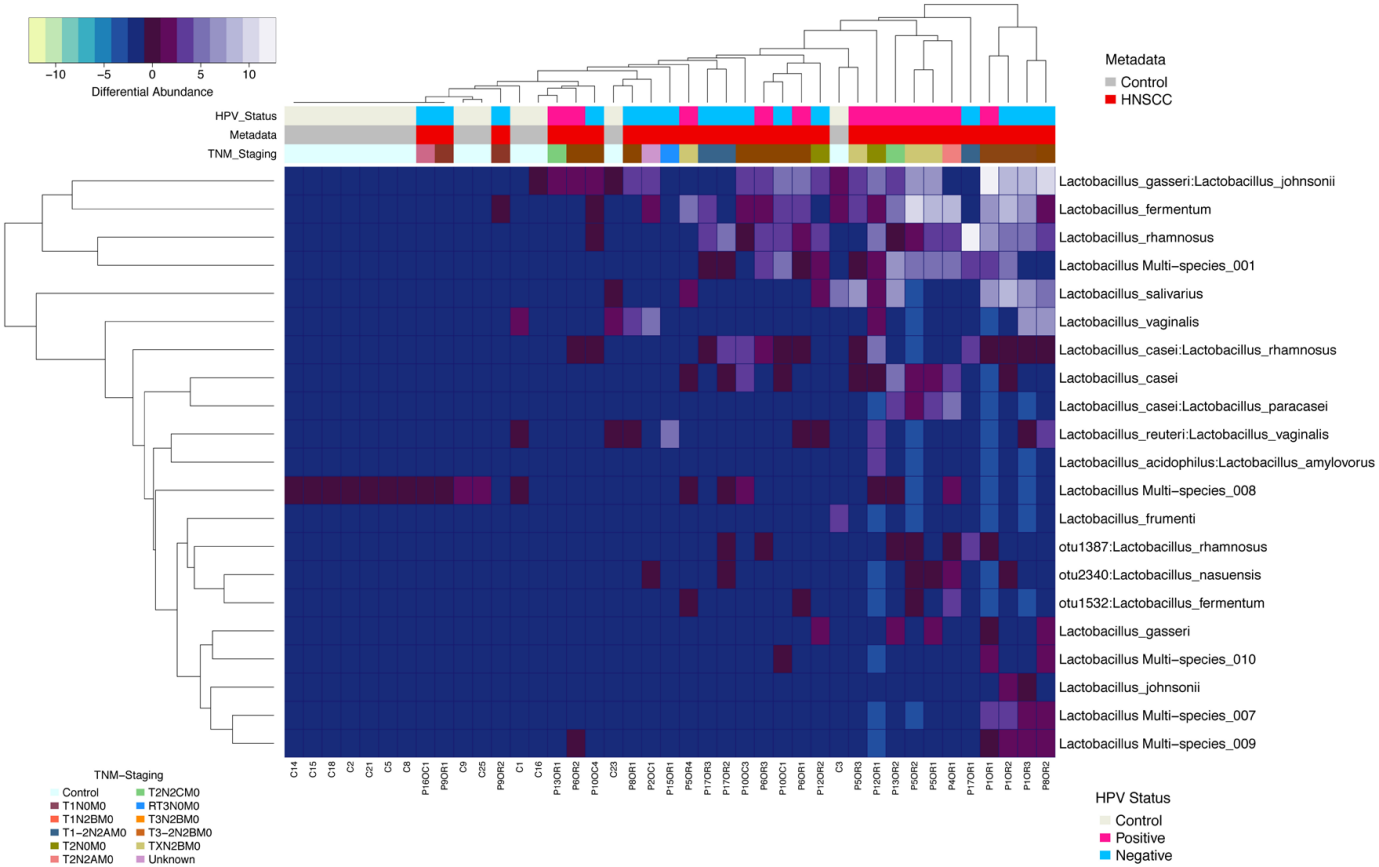
Heatmap differential abundance of significant (p<0.0001) Lactobacillus species’ OTUs in HNSCC when compared to JHU control samples with the variance stabilization method of QIIME’s 1.9.1 and DESeq2 normalization for data after logarithmic transformation, shows enrichment of *Lactobacillus gasseri:johnsonnii, Lactobacillus fermentum* and *Lactobacillus rhamnosus* with HNSCC samples A single *Lactobacillus* OTU with multiple ambiguous Resphera assignments (multi species 008) was more abundant in control samples.

Differentially enriched *Lactobacillus* species OTUs in HNSCC and JHU control samples were compared using the negative binomial Wald test for dispersion followed by variance stabilization. We found a significant association between *Lactobacillus gasseri:johnsonii*, *Lactobacillus fermentum* and *Lactobacillus rhamnosus* with HNSCC samples (p<0.0001). A single *Lactobacillus* OTU with multiple ambiguous Resphera assignments (multi species 008) was more abundant in control samples (Figure 5).

**Figure 6:**
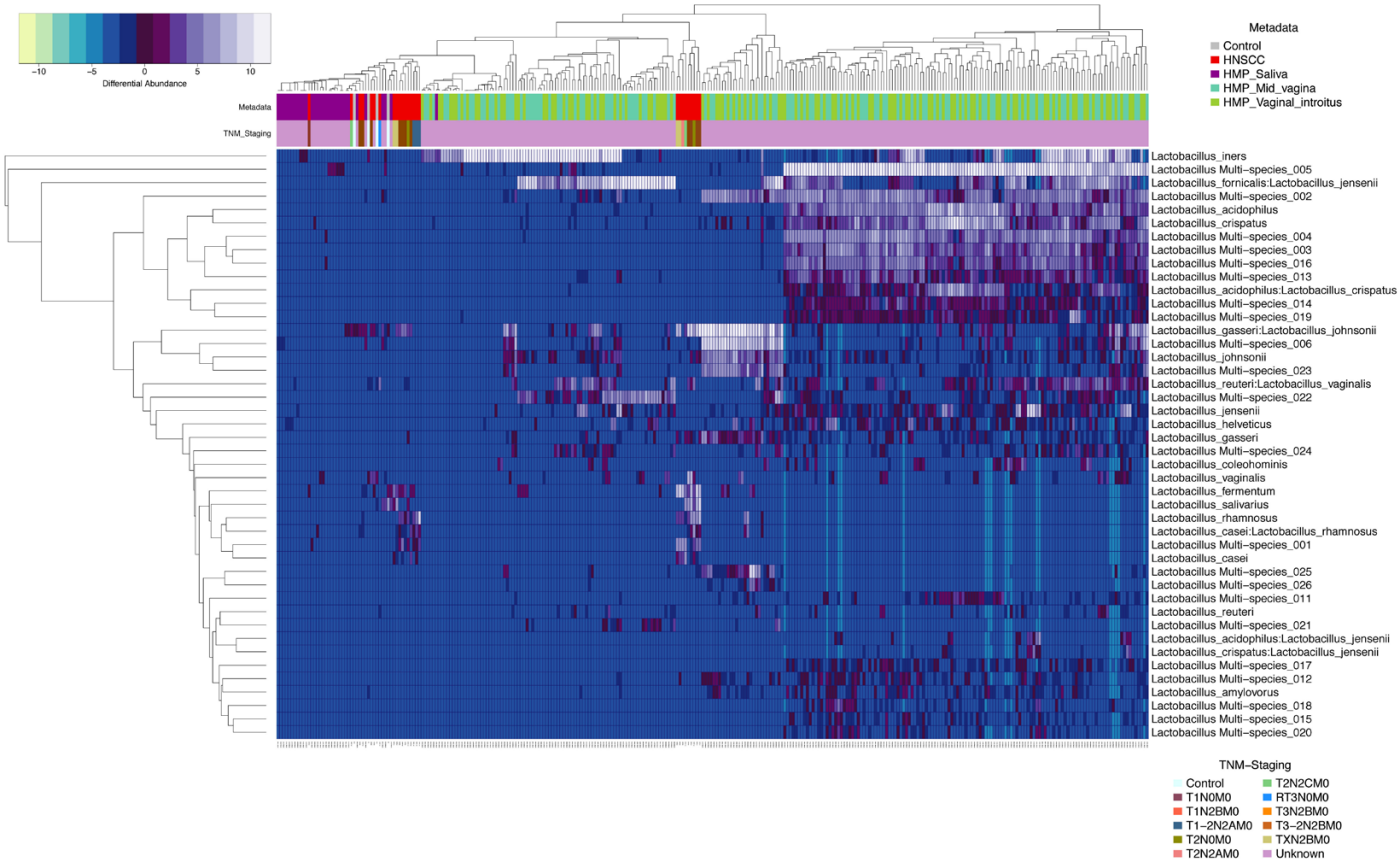
Heatmap differential abundance of significant (p<0.0001) Lactobacillus species’ OTUs in HNSCC when compared to JHU control samples and saliva, mid_vagina and vaginal_introitus samples from normal HMP participants with the variance stabilization method of QIIME’s 1.9.1 and DESeq2 normalization for data after logarithmic transformation, shows differential enrichment of *Lactobacillus_gasseri:Lactobacillus_johnsonnii, Lactobacillus vaginalis, Lactobacillus fermentum, Lactobacillus salivarius and Lactobacillus rhamnosus* in 34 HNSCC samples when compared to 25 normal JHU saliva samples, as well as 290 saliva samples and 249 vaginal samples from the HMP

We similarly compared significant (p<0.0001) differentially enriched *Lactobacillus* species OTUs in HNSCC and control samples from JHU and HMP saliva, mid-vagina and vaginal introitus from normal participants (Figure 6). This complex comparison shows that *Lactobacillus_gasseri:Lactobacillus_johnsonii*, *Lactobacillus vaginalis, Lactobacillus fermentum, Lactobacillus salivarius* and *Lactobacillus rhamnosus* were differentially enriched in 34 HNSCC samples when compared to 25 normal JHU saliva samples, as well as 290 saliva samples and 249 vaginal samples from the HMP.

Differentially enriched *Fusobacterium* species OTUs in HNSCC and control samples were also compared using the negative binomial Wald test for dispersion followed by variance stabilization. (Supplementary Figure 6A). We found a significant enrichment of *Fusobacterium nucleatum* and *Fusobacterium naviforme* in a subset of HNSCC samples. *Fusobacterium* species, such as *F. canifelium, F. nucleatum* and *F. naviforme* were differentially abundant across all samples. We also compared differentially enriched *Fusobacterium* species OTUs between HNSCC samples and saliva controls from JHU and HMP saliva, mid-vagina and introitus (Supplementary Figure 6B). This complex comparison revealed an enrichment of *Fusobacterium nucleatum* and *Fusobacterium naviforme* with specific HNSCC samples.

We observed differences in abundance of significant *Fusobacterium nucleatum* reads in a subset of HNSCC in comparison to normal oral microbiota of HNSCC samples and saliva controls from JHU and HMP saliva (Supplementary Figure 6C), The relative enrichment of *Fusobacterium_nucleatum* in HNSCC saliva is mostly seen in patients with larger tumors and nodal involvement (T3, N2) (Supplementary Figure 6D) and only in patients treated with surgery and chemo-radio-therapy when compared with patients only treated with surgical removal of the tumor (Supplementary Figure 6E).

The relative enrichment of significant OTUs 16S rRNA NGS reads of *Fusobacterium_nucleatum, Lactobacillus_gasseri:Lactobacillus_johnsonii*, and *Lactobacillus gasseri* in HNSCC saliva were compared by anatomic site (Supplementary Figure 7A), longitudinal sampling (Supplementary Figure 7B) and TNM stage (Supplementary Figure 7C). These three species are only concurrently seen in a subset of OPSCC patients: patients 5, 8, 10 and 13. *Fusobacterium_nucleatum*, and *Lactobacillus_gasseri:Lactobacillus_johnsonii*, were concurrently seen in more than half of the patients (8/17). The three species are only seen together in patients with larger tumors and nodal involvement (T2N2b or higher). We observed that *Lactobacillus_gasseri:Lactobacillus_johnsonii*, and *Fusobacterium_nucleatum* can be present in both Oral Squamous Cell Carcinoma (OSCC) and OPSCC patients. We also observed that *Lactobacillus_gasseri* is only enriched in some OPSCC patients and always together with *Lactobacillus_gasseri:Lactobacillus_johnsonii* enrichment. More importantly, the combined enrichment of *Lactobacillus_gasseri:Lactobacillus_johnsonii* and *Fusobacterium_nucleatum*, is almost exclusively seen in larger lesions (T3) with nodal involvement (N2b and above). These patterns of relative enrichment occur in a background of *Fusobacterium* species presence, as shown by PCR amplification of *Fusobacterium_spp* in Supplementary Figures 7D-7F.

### Longitudinal analyses of selected samples

**Figure 7:**
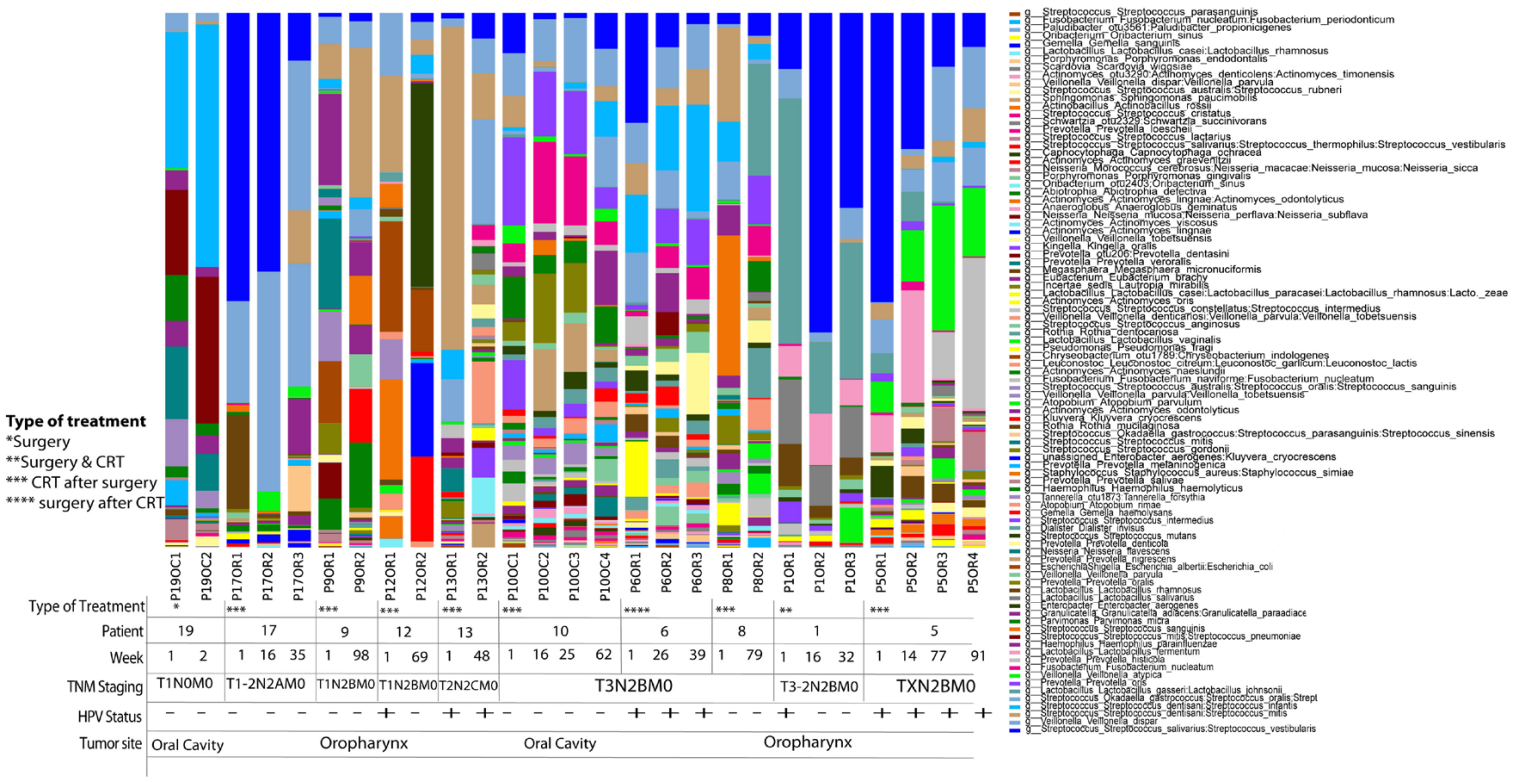

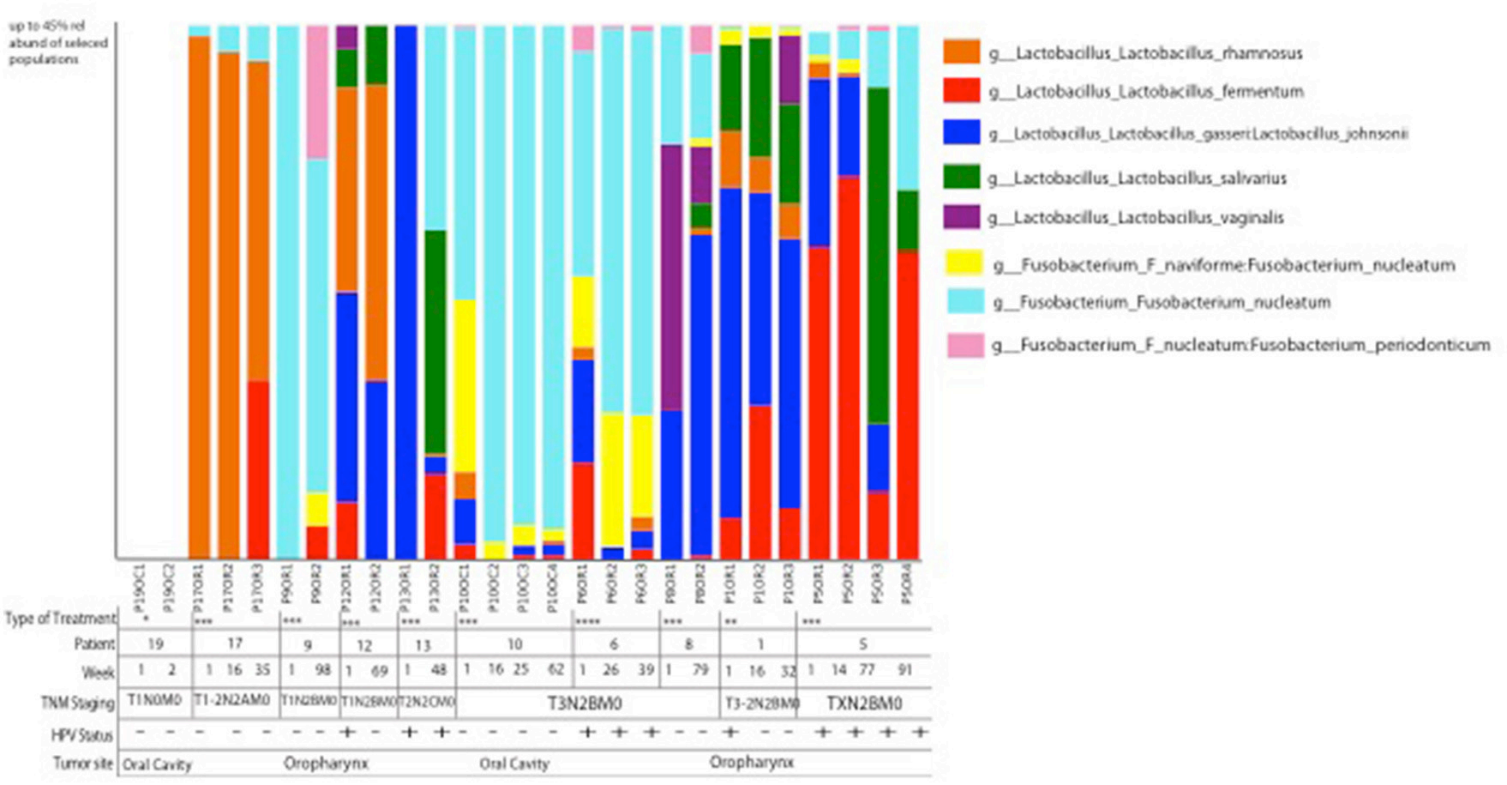
**(A)** Time-series analyses of HNSCC patients (n=11) for whom we had repeated saliva samples, according to both sampling sites, HPV status and TNM staging. The interval between sample collections ranged widely from 2 to 99 weeks, with a mean of 25.4 weeks, a median of 16.5 weeks and interquartile range of 25.4 weeks. Repeated samples were analyzed according to the type of treatment, HPV status and TNM staging based on relative abundance of OTUs. We found that community structure fluctuated by patient, but not significantly across all patients. **(B)** Relative abundance of Lactobacillus and Fusobacterium species in HNSCC patients (n=11) for whom we had repeated saliva samples, according to both sampling sites, HPV status and TNM staging. Bacterial communities were noticeably different between T1-2N2AM0 or lower and T3N2BM0 or higher TNM stage. While each patient had differentially abundant taxa between each time point, compared to each other overall, there was a decrease in Streptococcus as TNM stage progressed. Simultaneously, Lactobacillus_salivarius, Lactobacillus_fermentum, Lactobacillus_gasseri_johnsonii, and Lactobacillus_vaginalis’s OTUs increased in higher TNM stage categories across all patients (p<0.05).

We collected longitudinal post-treatment samples from a subset of 11 patients. We had two samples collected from six patients, three samples collected from three patients and four samples collected from four patients. The interval between sample collections ranged widely from 2 to 99 weeks, with a mean of 25.4 weeks, a median of 16.5 weeks and interquartile range of 25.4 weeks. Repeated samples were analyzed according to the type of treatment, HPV status and TNM staging based on relative abundance of OTUs. We found that community structure fluctuated by patient, but not significantly across all patients (Figure 7a). We did not observe significant longitudinal associations between community profiling and HPV status or TNM stage, probably due to small sample size and the wide range of intervals between repeated sampling. We did observe that while each patient had differentially abundant taxa between each time point, compared to each other overall, there was a decrease in Streptococcus as TNM stage progressed. Simultaneously, *Lactobacillus_salivarius, Lactobacillus_fermentum, Lactobacillus_gasseri_johnsonii*, and *Lactobacillus_vaginalis*’s OTUs increased in higher TNM stage categories across all patients (p<0.05) (Figure 7b).

## DISCUSSION

This is the first analysis of 16S rRNA NGS data that identifies commensal species from the vaginal flora, *Lactobacillus gasseri/johnsonii* and *Lactobacillus vaginalis*, in saliva from HPV positive and HPV negative oropharyngeal cancer patients. These commensal vaginal species were not observed in the combined 290 saliva samples of normal participants from the JHU and HMP cohorts. This study is also the first to identify *Fusobacterium nucleatum*, an oral cavity flora commensal bacterium linked to colon cancer, enriched in saliva from a subset of HNSCC patients with advanced tumors, when compared with controls. These unexpected results were confirmed using high-resolution microbiome profiling to the species level, of NGS data from a total of 583 clinical and HMP control samples from the oral and vaginal flora.

We found a significant presence of Lactobacillus OTUs from several different species in tumor when compared to control samples: Lactobacillus_gasseri:Lactobacillus_johnsonii, Lactobacillus vaginalis, Lactobacillus fermentum, Lactobacillus salivarius and Lactobacillus rhamnosus. Subset analyses of the longitudinal samples revealed that the abundance of Lactobacillus_gasseri:Lactobacillus_johnsonii, Lactobacillus fermentum, Lactobacillus salivarius and Lactobacillus rhamnosus was higher in oropharyngeal cancer patients. We also found increased abundance of Lactobacillus species with advanced T and N stages, in patients from whom we had collected saliva samples at different time points.

These results expand to the species level the findings of our prior report, which for the first time detected *Lactobacillus* in saliva from HNSCC patients [53]. As expected, the high-resolution microbiome profiling tools used in this report identified the presence of the same phyla we observed in our previous report: *Firmicutes, Actinobacteria, Bacteroidetes, Fusobacteria, and Proteobacteria*. Another group recently reported that *Actinomyces*, and its parent taxa up to the phylum level, were significantly depleted in HNSCC tumor tissue when compared to adjacent normal tissue [54]. They also reported that, *Parvimonas* was increased in HNSCC relative to normal tissue; *Actinobacteria* is differentially enriched in oral and oropharyngeal cancer tissue samples when compared to nasopharyngeal and larynx tissue samples; and that these differences were more pronounced among patients with more extensive disease, as measured by higher T-stage [54]. This same group also previously reported an association between microbiomic profiles and *MDR1* methylation in tissues from HNSCC patients when compared to normal tissues [55]. None of these reports discussed the presence of *Lactobacillus* in HNSCC tissue samples.

*Lactobacillus* has been associated with caries, hyposalivation or xerostomia, and has been observed in deep dentinal caries associated with pulpitis in adults [56]. However, their role is mainly protective. Lactobacilli inhibit binding of other bacteria to epithelial cells and produce lactic acid that kills or inhibits the growth of many other bacteria. Lactic acid blocks histone deacetylases, thereby enhancing gene transcription and DNA repair. Lactic acid induces autophagy in epithelial cells to degrade intracellular microorganisms and promote homeostasis [57].

For many decades, lactobacilli have been used as an effective probiotic therapy against several pathological conditions. *in vitro* and *in vivo* studies show that prolonged lactobacilli administration induces qualitative and quantitative modifications in the human gastrointestinal microbial ecosystem, with concurrent physiological and immunological changes that lead to improvement of different pathological conditions [58, 59].

Lactobacilli are the most abundant vaginal bacteria in women where they play a protective role. Lactobacilli are tolerated by vaginal epithelial cells and inhibit induction of pro-inflammatory cytokines. The normal microbial flora of the vagina plays an important role in preventing genital and urinary tract infections in women. The association between the vaginal microbiome and bacterial vaginosis (BV), an imbalance of the vaginal bacterial microbiota of unknown etiology, has been studied with different methods. Studies of the vaginal flora using NGS, 454-pyrosequencing, PCR and microbial isolation methods have yielded inconsistent results [60]. The prevalence of *Lactobacillus crispatus, Lactobacillus jensenii*, and *Lactobacillus gasseri* is significantly higher in the vaginal flora of healthy women when compared to women with BV [61]. The vaginal flora in women with BV changes from a *Lactobacillus*-dominant vaginal microbiota to an anaerobic and facultative bacterial dominance. It is only in those women with a BV diagnosis in which the identified bacteria are atypical and persist that BV may be a clinical problem requiring intervention [62]. Analysis of the bacterial communities by 16S rRNA amplicon sequencing revealed two clusters in the BV negative women, dominated by either *Lactobacillus iners* or *Lactobacillus crispatus* and three distinct clusters in the BV positive women. BV positive subjects showed cluster profiles that were relatively high in bacterial species diversity and dominated by anaerobic species, including Gardnerella vaginalis, and those belonging to the Families of *Lachnospiraceae* and *Leptotrichiaceae* *[63]*.

The normal microbial flora of the vagina also seems to differ across race and cultural divides. Black women are more likely to be colonized by *L. iners* than the other *Lactobacillus* species. In contrast, healthy women of European ancestry are more likely to be colonized with three health-associated *Lactobacillus* species, namely *L. crispatus*, *L. jensenii* and *L. gasseri*, and exhibited significantly lower bacterial diversity. *Lactobacillus gasseri* was found to be negatively associated with BV in Chinese women [64]. *Lactobacillus johnsonii, Lactobacillus gasseri* and *Lactobacillus vaginalis* strains have been isolated from women in Brazil with or without BV, with an intermediate frequency in the two groups [65].

Together these data suggest that the presence of *Lactobacillus gasseri/johnsonii* and *Lactobacillus vaginalis* in saliva from oropharyngeal patients may be due to the transmission of commensal bacterial from normal vaginal to the oral flora during oral sex. If this finding is validated in other cohorts, potential interactions between *Lactobacillus gasseri/johnsonii* and *Lactobacillus vaginalis* in saliva with the persistence and eventual carcinogenesis of high risk HPV types in oropharyngeal cancer should be explored. Their role in the apparent difference in oropharyngeal cancer rates between Black and Non-Latino White men should also be explored.

This study is also the first to identify *Fusobacterium nucleatum* enriched in a subset of saliva samples from HNSCC patients, when compared with controls. Interestingly, we also observed a highly significant depletion of bacterial species closely related to *Fusobacterium nucleatum* in saliva from in HNSCC patients. *Fusobacterium periodonticum, Leptotrichia trevisanii, Leptotrichia hofstadii*, and *Leptotrichia buccalis* are enriched in saliva control samples from the JHU and HMP cohorts. Our PCR results for *Fusobacterium*_spp suggest a ubiquitous presence of the *Fusobacterium* family in HNSCC.

*Fusobacterium nucleatum* is one of the whole *Fusobacterium* species (Pan-fusobacterium) that are abundant in colorectal carcinoma (CRC) tissue compared with adjacent normal mucosa [66, 67]. *Fusobacterium* enrichment is associated with a subset of tumors that exhibit well-characterized molecular hallmarks of CRC: CpG island methylator phenotype (CIMP), Microsatellite Instability (MSI), hMLH1 methylation positivity and high mutation burden [68]. It is not clear if *Fusobacterium nucleatum* is a driver of tumorigenesis or a well- positioned passenger. A potential oncogenic mechanism has been proposed: *Fusobacterium nucleatum* may promote colorectal carcinogenesis by modulating E-cadherin/β-catenin signaling via its FadA adhesion molecule. FadA binds to E-cadherin, activates β-catenin signaling, and differentially regulates inflammatory and oncogenic responses [69]. Binding of FadA to E-cadherin on the cell surface mediates attachment and invasion of *Fusobacterium nucleatum* into CRC epithelial cells, inhibiting their tumor-suppressor activity and activating b-catenin signaling. *Fusobacterium nucleatum* then invades the cell via internalization of E-cadherin by Clathrin and stimulates expression of inflammatory genes [70]. As a result, b-catenin phosphorylation is decreased, resulting in increased b-catenin in the cytoplasm and translocation into the nucleus where it modulates increased expression of *LEF*, *TCF*, *NF-kb*, and other oncogenes. On the other hand, *Fusobacterium nucleatum* does not exacerbate colitis, enteritis, or inflammation- associated intestinal carcinogenesis, which is contrary to the role of other colon cancer-associated bacteria [71]. *Fusobacterium nucleatum* alone may not be a driver of colorectal tumorigenesis, but may contribute to the development of colorectal cancer by synergistic associations with other bacteria or even possibly with fungi and viruses [72]. Together, the data suggest that *Fusobacterium nucleatum* may generate, in a subset of patients, a proinflammatory microenvironment through recruitment of tumor-infiltrating immune cells that is conducive for colorectal neoplasia progression.

The microbiome of the oral cavity is the second most diverse one of the human body after the gut [52]. The oral microbiome, which can cause both oral and systemic disease, grows in an ecosystem of biofilms throughout the oral cavity, existing in a balanced immunoinflammatory state with the host that maintains health when in equilibrium [73]. However, certain species, such as *Porphyromonas gingivalis*, can disrupt this equilibrium, resulting in dysbiotic host-microbiota interactions [74]. *Fusobacterium nucleatum* is considered to be a key oral bacterium in recruiting periodontal pathogens into subgingival dental plaque [75].

There is emerging evidence of associations between malignant oral lesions with chronic periodontitis, as well as chronic abundance of *Porphyromonas gingivalis*, and *Fusobacterium nucleatum*, among other microbes [76]. OSCC surfaces have been reported to harbor significantly higher levels of *Porphyromonas* and *Fusobacterium* compared with contiguous healthy mucosa [77]. However, a causal role for oral microbiota in OSCC or OPSCC is just beginning to be delineated. *Porphyromonas gingivalis* induces the expression of the B7-H1 and B7-DC receptors in primary OSCC cells, which are upregulated in a variety of cancers and contribute to chronic inflammation [78]. Chronic infection with *Porphyromonas gingivalis* and *Fusobacterium nucleatum* has been recently shown to promote tongue tumors in a murine model via direct interaction with oral epithelial cells, leading to upregulation of the IL-6-STAT3 pathway in a TLR2-dependent manner [79].

The oral microbiome metabolism is not well studied [80]. Early studies suggest that that the main carbon metabolic pathways, such as the Embden-Meyerhof-Parnas (EMP) pathway, the pentose phosphate (PP) pathway, and the tri-carbonic acid (TCA) cycle, as well as amino acid metabolism-related pathways, are in operation in the oral biofilm *in vivo* and exhibit similar characteristics to those of *Streptococcus* and *Actinomyces* [81, 82]. Dysregulation of these main carbon metabolic pathways have been correlated to dental caries, periodontal disease and oral cancer in small studies [83–85]. The following five saliva metabolomic biomarkers can discriminate from early HNSCC patients from controls: propionylcholine, N-Acetyl-L-phenylalanine, sphinganine, phytosphingosine, and S-carboxymethyl-L-cysteine [86]. Their association to members of the saliva microbiome has not been studied. There is contradictory metabolomics evidence supporting the pathogenic role of *Fusobacterium nucleatum* in the oral cavity. Some studies have observed that *Fusobacterium nucleatum* upregulates the expression of lysine degradation pathway leading to butyrate production in periodontitis patients [87]. However, the routes of butyrate production may differ by Fusobacteria strain or substrains [88]. *Fusobacterium nucleatum* can be subdivided into five subspecies. Virulence genes in the W1481 subspecies genome may provide a strong defense mechanism that might enable it to colonize and survive within the host by evading immune surveillance [89].

Further studies are needed to determine the role of *Lactobacillus gasseri/johnsonii*, *Lactobacillus vaginalis* and *Fusobacterium nucleatum* in OPSCC and OSCC pathogenesis in diverse populations and their possible uses for screening, diagnostic and chemoprevention strategies with probiotic therapy. Gut microbiome has been associated with response to immune checkpoint blockade [90]. While immune checkpoint inhibitors are emerging as a promising treatment modality for HNSCC [91], the oral microbiome should be investigated for potential interactions with immune therapy.

## MATERIALS AND METHODS

### Johns Hopkins Hospitals cohort

The Discovery cohort patients selected for this study were nested within a longitudinal cohort study of 787 patients who presented with histopathologically confirmed HSNCC (this includes patients who presented for treatment of a recurrence after primary treatment at an outside hospital) to the outpatient clinic of the Johns Hopkins Hospital in Baltimore, Maryland from 2000 to 2011. Patients were included if they had at least one pre-treatment salivary sample and consented for the study. All patients had undergone treatment with curative intent. Patients were consented for this study under the molecular surveillance clinical research protocol. The study protocol was approved by the institutional review board of the Johns Hopkins Hospital, as well as by the JHU Institutional Review Board. Written informed consent was obtained from all patients. Once consented, patients are tracked by our patient coordinators, who working in tandem with our surgeons, use the same sample collection and processing protocol that have historically been used in the Head and Neck Cancer Tumor Bank (HAND) to collect and process samples.

Samples were collected between 2000 and 2011 and stored in the JHU Head and Neck Cancer Research Division’s Tumor Bank, from where they were randomly selected for this study. Saliva was collected from 44 patients: 25 patients with no history of cancer and 19 HNSCC patients. Longitudinal saliva samples were collected from 58% of the HNSCC patients, totaling 62 samples. None of the patients were treated with antibiotics during a six-month period prior to sample collection. We eliminated 3 samples from 2 patients (2 OSCC and 1 OPSCC), 2 of them with unknown HPV status, as well as an HPV positive OSCC saliva sample.

The analyses presented here are based on a total of 59 saliva samples acquired from 42 patients; of these, 34 saliva samples corresponded to 17 HNSCC patients and 25 saliva samples corresponded to 25 controls without cancer, which also had negative smoking and drinking histories. Most of the HNSCC patients were OPSCC (58%). There was no difference in median age (66 and 62 years) or percentage of male patients (67% and 77%) between OSCC and OPSCC patients. Forty-seven percent (47%) of the HNSCC, all of them OPSCC patients, were HPV positive. Most OPSCC patients (64%) were HPV positive. Most HNSCC patients were former smokers or never smokers: OPSCC (72%) and OSCC (63%). Similarly, most HNSCC patients were occasional drinkers or non-drinkers: OPSCC (72%) and OSCC (63%). Approximately half of the patients were diagnosed with T1 or T2 stage tumors: OSCC (50%) and OPSCC (55%). Most patients had nodal involvement: OSCC (75%) and OPSCC (73%). None of the patients had known metastasis. All the OPSCC were treated with surgery and chemoradiation, compared to only 25% of the OSCC. Most of the OSCC patients (63%) only required surgical treatment (Supplementary Table 2). Ten patients provided between 1 to 3 longitudinal, post-treatment samples. The age of these patients ranged from 43 to 67 years of age, with a median 61 years. Most patients were males (80%), OPSCC (80%), T1-T2 stage (70%), non-smokers (70%), treated with surgery, followed by chemo-radiation (70%) treatment (Supplementary Table 3).

### Human microbiome project cohort

We obtained quality-controlled 16S rRNA sequence datasets from the Human Microbiome Project Data Analysis and Coordination Center (HMPDACC; public-ftp.hmpdacc.org [52, 92]), generated by sequencing of the 16S rRNA V3–V5 hypervariable region on the Roche-454 FLX Titanium instrument for a total of 537 saliva, mid-vagina, and vaginal introitus samples. In total, 514 of the 537 HMP samples (96%) downloaded for this project had at least 2,500 high-quality V3V5 sequences (≥ 200bp) and were subsequently used for downstream analysis including healthy human saliva (n=265), mid-vagina (n=128), and vaginal introitus (n=121) samples. These 514 samples were obtained from 154 unique participants: saliva (n=154), mid-vagina (n=79), and vaginal introitus (n=73), most of whom provided two longitudinal samples.

### DNA extraction

Microdissected tissues and saliva (2mL) samples were centrifuged and the pellets were digested with 1% SDS and 50 μg/mL proteinase K (Boehringer, Mannheim, Germany) at 48°C overnight extracted with phenol/chloroform, precipitated in 100% ethanol, centrifuged at 5100 rpm for 45 minutes, washed in 70% ethanol twice, dissolved in LoTE buffer (10mM TRIS hydrochloride, 1mM EDTA buffer, pH 8), and stored at −20°C [93].

### Creation of the 16S rRNA V3-V5 amplicon library

An amplicon library from individual samples was created by PCR amplification with unique barcoded primers of the 16S rRNA V3-V5 gene region, using the 357F/926R primer set. We used 14 different barcode sequences and the linker primer sequence CCGTCAATTCMTTTRAGT to analyze the 16S rRNA V3–V5 hypervariable 16S rRNA gene region.

### DNA sequencing and bioinformatics analyses

Sequencing of the multiplexed amplified fragments was performed on the Roche/454 GS Junior pyrosequencing platform. Bioinformatics preprocessing steps included quality filtering, error-correction, and chimera removal. Briefly, reads were de-multiplexed using 5’ barcodes, trimmed of forward and reverse primer sequences, filtered for length and quality, and corrected for homopolymer errors. High quality reads were selected for analysis and reads with unknown bases (“N”) were discarded.

Sequences underwent strict quality and size filtering, removing reads shorter than 150bp, as well as those with mismatches and poor quality scores. Sequences were then error-corrected using the Acacia tool, followed by de novo chimera detection with the UCHIME program, and screening for chloroplast contaminant sequences, as previously described [53].

High-quality 16S rRNA amplicon sequences passing preprocessing were submitted to Resphera Insight (Baltimore, MD, www.respherabio.com) for high-resolution taxonomic identification of bacterial species [50, 51, 94–96]. The Insight methodology relies on a manually curated 16S rRNA database with over 11,000 bacterial species and a hybrid global-local alignment strategy to provide accurate species-level context for 16S rRNA microbiome profiling studies.

After considering the raw count data in full above, subsample analysis of each community was performed to an equivalent depth, in this case, 3,400 sequences per sample for the comparisons between HNSCC and control saliva data. When comparisons were done with HPM datasets, we used 2,500 sequences per sample to avoid eliminating samples for analyses. All results are therefore based on the subsampled data, which mitigates biases due to differences in sampling depth.

An OTU network was generated using QIIME [97] and imported to Cytoscape [98] based on OTUs that changed significantly in abundance (p<0.05) as result of a maximum likelihood statistical significance tests. The selected OTUs were plotted choosing nodes from the OTU network and sorting edges interaction by the four different sample types: normal, HPV negative OSCC, HPV negative OPSCC and HPV positive OPSCC. Additionally, we represented the taxonomy of taxa at the genus-level through pie charts at each of the four sample types.

### Quantitative PCR

The 7900HT real time PCR system was used to perform quantitative PCR for HPV-16 *E6* and *E7* and *B*-actin. Specific primers and probes have been designed to amplify the *E6* and *E7* regions of HPV type 16: HPV-16 *E6* forward primer, 5’-TCAGGACCCACAGGAGCG-3’; HPV-16 *E6* reverse primer, 5’-CCTCACGTCGCAGTAACTGTTG-3’, HPV-16 *E6* TaqMan probe, 5’-(FAM)-CCCAGAAAGTTACCACAGTTATGCACAGAGCT-(TAMRA)-3’, HPV-16 *E7* forward primer, 5’-CCGGACAGAGCCCATTACAA-3’, HPV-16 *E7* reverse primer, 5’-CGAATGTCTACGTGTGTGCTTTG-3’, HPV-16 *E7* TaqMan probe, 5’-(FAM)-CGCACAACCGAAGCGTAGAGTCACACT-(TAMRA)-3’. A housekeeping gene (*B-actin*) were run in parallel with HPV-16 *E6* and *E7* to standardize the input DNA: *B*-actin forward primer, 5’-TCACCCACACTGTGCCCATCTACGA-3’, *B*-actin reverse primer, 5’-CAGCGGAACCGCTCATTGCCAATGG-3’, *B*-actin TaqMan probe, 5’-(FAM)-ATGCCCTCCCCCATGCCATCCTGCGT-(TAMRA)-3’. The 7900HT real time PCR system was also used to perform quantitative PCR for *Fusobacterium_spp.* and *B*-actin. We used previously designed primers and probes to amplify *Fusobacterium_spp* [99]: *Fusobacterium_spp* forward primer, 5’- GGATTTATTGGGCGTAAAGC -3’; *Fusobacterium_spp* reverse primer, 5’- GGCATTCCTACAAATATCTACGAA -3’; *Fusobacterium_spp* TaqMan probe, 5’-(FAM)- CTCTACACTTGTAGTTCCG -(TAMRA)-3’.

All samples were run in triplicate. The CaSki (American Type Culture Collection, Manassas, VA) cell line was used to develop standard curves for the HPV viral copy number as it is known to have 600 copies/genome equivalent. Standard curves for HPV-16 *E6* and *E7* were developed by using DNA extracted from CaSki cells, serially diluted into 50 ng, 5 ng, 0.5 ng, 0.05 ng, and 0.005 ng. A standard curve was also developed for the housekeeping gene *B-actin* (2 copies/genome), using the same serial dilutions of CaSki DNA. Saliva samples with > 0 copy/genome were considered as HPV positive. No statistical correlation was attempted due to modest sample size.

### Statistical analysis

For each group comparison, significance tests were computed including the maximum likelihood statistical significance tests that determine whether OTU presence/absence is associated with a category in the metadata. QIIME [97] was used to run the R interface package of DESeq2 for the negative binomial Wald test and Variance Stabilizing Transformations. These procedures diminish the large variation between count data across diverse samples providing a robust method of comparison between samples. Reported fold changes for specific species reflect the ratio of mean relative abundance in cancer patients to the mean of controls. The relative group variance homogeneity was verified with the function ‘betadisper’ also in the “vegan” package. Richness box and whisker plots were calculated using both vegan [100] and Phyloseq [101] R packages.

QIIME was also used to generate clustering with heatmaps and estimate alpha-diversity metrics including raw number of OTUs per sample, Chao1 estimator, Shannon entropy, Non-Metric dimensional scaling, and Bray-Curtis distance metric. To better understand the bacterial OTU diversity in our cohorts, we compared the alpha rarefaction curves between normal and HNSCC samples according to the Chao 1 richness estimator. The Chao1 index estimates total species richness based on all species actually discovered, including species not present in any sample. This approach uses the numbers of singletons (single appearance) and doubletons (that appeared twice) to estimate the number of missing species due to undetected species information on low frequency counts.

## SUPPLEMENTARY MATERIALS FIGURES AND TABLES







## References

[1] Blaser MJ (2008). Understanding microbe-induced cancers. Cancer Prev Res (Phila).

[2] Ohtani N (2015). Microbiome and cancer. Semin Immunopathol.

[3] Maldonado-Contreras A, Goldfarb KC, Godoy-Vitorino F, Karaoz U, Contreras M, Blaser MJ, Brodie EL, Dominguez-Bello MG (2011). Structure of the human gastric bacterial community in relation to Helicobacter pylori status. ISME J.

[4] Lee IO, Kim JH, Choi YJ, Pillinger MH, Kim SY, Blaser MJ, Lee YC (2010). Helicobacter pylori CagA phosphorylation status determines the gp130-activated SHP2/ERK and JAK/STAT signal transduction pathways in gastric epithelial cells. J Biol Chem.

[5] Brawner KM, Morrow CD, Smith PD (2014). Gastric microbiome and gastric cancer. Cancer J.

[6] Kerr AR (2015). The oral microbiome and cancer. J Dent Hyg.

[7] Michaud DS, Izard J (2014). Microbiota, oral microbiome, and pancreatic cancer. Cancer J.

[8] Narayanan V, Peppelenbosch MP, Konstantinov SR (2014). Human fecal microbiome-based biomarkers for colorectal cancer. Cancer Prev Res (Phila).

[9] Weir TL, Manter DK, Sheflin AM, Barnett BA, Heuberger AL, Ryan EP (2013). Stool microbiome and metabolome differences between colorectal cancer patients and healthy adults. PLoS One.

[10] Geng J, Fan H, Tang X, Zhai H, Zhang Z (2013). Diversified pattern of the human colorectal cancer microbiome. Gut Pathog.

[11] Zambirinis CP, Pushalkar S, Saxena D, Miller G (2014). Pancreatic cancer, inflammation, and microbiome. Cancer J.

[12] Seki E (2014). Microbiome-obesity-liver cancer interaction: senescence of hepatic stellate cells and bile acids play new roles. Gastroenterology.

[13] Cook MB, Dawsey SM, Diaw L, Blaser MJ, Perez-Perez GI, Abnet CC, Taylor PR, Albanes D, Virtamo J, Kamangar F (2010). Serum pepsinogens and Helicobacter pylori in relation to the risk of esophageal squamous cell carcinoma in the alpha-tocopherol, beta-carotene cancer prevention study. Cancer Epidemiol Biomarkers Prev.

[14] Blaser MJ (2010). Helicobacter pylori and esophageal disease: wake-up call?. Gastroenterology.

[15] Plottel CS, Blaser MJ (2011). Microbiome and malignancy. Cell Host Microbe.

[16] Wynendaele E, Verbeke F, D'Hondt M, Hendrix A, Van De Wiele C, Burvenich C, Peremans K, De Wever O, Bracke M, De Spiegeleer B (2015). Crosstalk between the microbiome and cancer cells by quorum sensing peptides. Peptides.

[17] Virchow R (1989). Cellular pathology. As based upon physiological and pathological histology. Lecture XVI--Atheromatous affection of arteries. 1858. Nutr Rev.

[18] Shalapour S, Karin M (2015). Immunity, inflammation, and cancer: an eternal fight between good and evil. J Clin Invest.

[19] Grivennikov SI, Greten FR, Karin M (2010). Immunity, inflammation, and cancer. Cell.

[20] Vetizou M, Pitt JM, Daillere R, Lepage P, Waldschmitt N, Flament C, Rusakiewicz S, Routy B, Roberti MP, Duong CP, Poirier-Colame V, Roux A, Becharef S (2015). Anticancer immunotherapy by CTLA-4 blockade relies on the gut microbiota. Science.

[21] Lalla RV, Bowen J, Barasch A, Elting L, Epstein J, Keefe DM, McGuire DB, Migliorati C, Nicolatou-Galitis O, Peterson DE, Raber-Durlacher JE, Sonis ST, Elad S (2014). MASCC/ISOO clinical practice guidelines for the management of mucositis secondary to cancer therapy. Cancer.

[22] Sonis ST, Elting LS, Keefe D, Peterson DE, Schubert M, Hauer-Jensen M, Bekele BN, Raber-Durlacher J, Donnelly JP, Rubenstein EB, Mucositis Study Section of the Multinational Association for Supportive Care in C, International Society for Oral O (2004). Perspectives on cancer therapy-induced mucosal injury: pathogenesis, measurement, epidemiology, and consequences for patients. Cancer.

[23] Shalapour S, Font-Burgada J, Di Caro G, Zhong Z, Sanchez-Lopez E, Dhar D, Willimsky G, Ammirante M, Strasner A, Hansel DE, Jamieson C, Kane CJ, Klatte T (2015). Immunosuppressive plasma cells impede T-cell-dependent immunogenic chemotherapy. Nature.

[24] Vande Voorde J, Balzarini J, Liekens S (2014). An emerging understanding of the Janus face of the human microbiome: enhancement versus impairment of cancer therapy. J Antimicrob Chemother.

[25] Plottel CS (2014). From the guest editor: beyond symbiosis: a cancer-centric view of the microbiome. Cancer J.

[26] Toomer KH, Chen Z (2014). Autoimmunity as a double agent in tumor killing and cancer promotion. Front Immunol.

[27] Takeda K, Akira S (2005). Toll-like receptors in innate immunity. Int Immunol.

[28] Abreu MT (2010). Toll-like receptor signalling in the intestinal epithelium: how bacterial recognition shapes intestinal function. Nat Rev Immunol.

[29] Cheesman SE, Neal JT, Mittge E, Seredick BM, Guillemin K (2011). Epithelial cell proliferation in the developing zebrafish intestine is regulated by the Wnt pathway and microbial signaling via Myd88. Proc Natl Acad Sci U S A.

[30] Brubaker SW, Bonham KS, Zanoni I, Kagan JC (2015). Innate immune pattern recognition: a cell biological perspective. Annu Rev Immunol.

[31] Bultman SJ (2014). Emerging roles of the microbiome in cancer. Carcinogenesis.

[32] Ferris RL (2015). Immunology and Immunotherapy of Head and Neck Cancer. J Clin Oncol.

[33] Guo T, Califano JA (2015). Molecular biology and immunology of head and neck cancer. Surg Oncol Clin N Am.

[34] Reed AL, Califano J, Cairns P, Westra WH, Jones RM, Koch W, Ahrendt S, Eby Y, Sewell D, Nawroz H, Bartek J, Sidransky D (1996). High frequency of p16 (CDKN2/MTS-1/INK4A) inactivation in head and neck squamous cell carcinoma. Cancer Res.

[35] Koch WM, Lango M, Sewell D, Zahurak M, Sidransky D (1999). Head and neck cancer in nonsmokers: a distinct clinical and molecular entity. Laryngoscope.

[36] Gillison ML, Koch WM, Capone RB, Spafford M, Westra WH, Wu L, Zahurak ML, Daniel RW, Viglione M, Symer DE, Shah KV, Sidransky D (2000). Evidence for a causal association between human papillomavirus and a subset of head and neck cancers. J Natl Cancer Inst.

[37] Tokumaru Y, Yamashita K, Osada M, Nomoto S, Sun DI, Xiao Y, Hoque MO, Westra WH, Califano JA, Sidransky D (2004). Inverse correlation between cyclin A1 hypermethylation and p53 mutation in head and neck cancer identified by reversal of epigenetic silencing. Cancer Res.

[38] Ha PK, Califano JA (2006). Promoter methylation and inactivation of tumour-suppressor genes in oral squamous-cell carcinoma. Lancet Oncol.

[39] Agrawal N, Frederick MJ, Pickering CR, Bettegowda C, Chang K, Li RJ, Fakhry C, Xie TX, Zhang J, Wang J, Zhang N, El-Naggar AK, Jasser SA (2011). Exome sequencing of head and neck squamous cell carcinoma reveals inactivating mutations in NOTCH1. Science.

[40] Poeta ML, Manola J, Goldwasser MA, Forastiere A, Benoit N, Califano JA, Ridge JA, Goodwin J, Kenady D, Saunders J, Westra W, Sidransky D, Koch WM (2007). TP53 mutations and survival in squamous-cell carcinoma of the head and neck. N Engl J Med.

[41] Guerrero-Preston R, Michailidi C, Marchionni L, Pickering CR, Frederick MJ, Myers JN, Yegnasubramanian S, Hadar T, Noordhuis MG, Zizkova V, Fertig E, Agrawal N, Westra W (2014). Key tumor suppressor genes inactivated by "greater promoter" methylation and somatic mutations in head and neck cancer. Epigenetics.

[42] Sun W, Gaykalova DA, Ochs MF, Mambo E, Arnaoutakis D, Liu Y, Loyo M, Agrawal N, Howard J, Li R, Ahn S, Fertig E, Sidransky D (2014). Activation of the NOTCH pathway in head and neck cancer. Cancer Res.

[43] Gaykalova DA, Manola JB, Ozawa H, Zizkova V, Morton K, Bishop JA, Sharma R, Zhang C, Michailidi C, Considine M, Tan M, Fertig EJ, Hennessey PT (2015). NF-kappaB and stat3 transcription factor signatures differentiate HPV-positive and HPV-negative head and neck squamous cell carcinoma. Int J Cancer.

[44] Cancer Genome Atlas Network (2015). Comprehensive genomic characterization of head and neck squamous cell carcinomas. Nature.

[45] Hamady M, Knight R (2009). Microbial community profiling for human microbiome projects: Tools, techniques, and challenges. Genome Res.

[46] Wang L, Ganly I (2014). The oral microbiome and oral cancer. Clin Lab Med.

[47] Jumpstart Consortium Human Microbiome Project Data Generation Working Group (2012). Evaluation of 16S rDNA-based community profiling for human microbiome research. PLoS One.

[48] Ahn J, Sinha R, Pei Z, Dominianni C, Wu J, Shi J, Goedert JJ, Hayes RB, Yang L (2013). Human gut microbiome and risk for colorectal cancer. J Natl Cancer Inst.

[49] Ahn J, Yang L, Paster BJ, Ganly I, Morris L, Pei Z, Hayes RB (2011). Oral microbiome profiles: 16S rRNA pyrosequencing and microarray assay comparison. PLoS One.

[50] Daquigan N, Grim CJ, White JR, Hanes DE, Jarvis KG (2016). Early Recovery of Salmonella from Food Using a 6-Hour Non-selective Pre-enrichment and Reformulation of Tetrathionate Broth. Front Microbiol.

[51] White JR, Drewes J, Sears CL (2016). High-resolution microbiome profiling and meta-analysis yields insight into microbial consortia associated with colorectal cancer. Cancer Research.

[52] Human Microbiome Project Consortium (2012). Structure, function and diversity of the healthy human microbiome. Nature.

[53] Guerrero-Preston R, Godoy-Vitorino F, Jedlicka A, Rodriguez-Hilario A, Gonzalez H, Bondy J, Lawson F, Folawiyo O, Michailidi C, Dziedzic A, Thangavel R, Hadar T, Noordhuis MG (2016). 16S rRNA amplicon sequencing identifies microbiota associated with oral cancer, Human Papilloma Virus infection and surgical treatment. Oncotarget.

[54] Wang H, Funchain P, Bebek G, Altemus J, Zhang H, Niazi F, Peterson C, Lee WT, Burkey BB, Eng C (2017). Microbiomic differences in tumor and paired-normal tissue in head and neck squamous cell carcinomas. Genome Med.

[55] Bebek G, Bennett KL, Funchain P, Campbell R, Seth R, Scharpf J, Burkey B, Eng C (2012). Microbiomic subprofiles and MDR1 promoter methylation in head and neck squamous cell carcinoma. Hum Mol Genet.

[56] Badet C, Thebaud NB (2008). Ecology of lactobacilli in the oral cavity: a review of literature. Open Microbiol J.

[57] Witkin SS, Linhares IM (2017). Why do lactobacilli dominate the human vaginal microbiota?. BJOG.

[58] Di Cerbo A, Palmieri B, Aponte M, Morales-Medina JC, Iannitti T (2016). Mechanisms and therapeutic effectiveness of lactobacilli. J Clin Pathol.

[59] Goldenberg JZ, Lytvyn L, Steurich J, Parkin P, Mahant S, Johnston BC (2015). Probiotics for the prevention of pediatric antibiotic-associated diarrhea. Cochrane Database Syst Rev.

[60] van de Wijgert JH, Borgdorff H, Verhelst R, Crucitti T, Francis S, Verstraelen H, Jespers V (2014). The vaginal microbiota: what have we learned after a decade of molecular characterization?. PLoS One.

[61] Tamrakar R, Yamada T, Furuta I, Cho K, Morikawa M, Yamada H, Sakuragi N, Minakami H (2007). Association between Lactobacillus species and bacterial vaginosis-related bacteria, and bacterial vaginosis scores in pregnant Japanese women. BMC Infect Dis.

[62] Nasioudis D, Linhares IM, Ledger WJ, Witkin SS (2017). Bacterial vaginosis: a critical analysis of current knowledge. BJOG.

[63] Dols JA, Molenaar D, van der Helm JJ, Caspers MP, de Kat Angelino-Bart A, Schuren FH, Speksnijder AG, Westerhoff HV, Richardus JH, Boon ME, Reid G, de Vries HJ, Kort R (2016). Molecular assessment of bacterial vaginosis by Lactobacillus abundance and species diversity. BMC Infect Dis.

[64] Yan DH, Lu Z, Su JR (2009). Comparison of main lactobacillus species between healthy women and women with bacterial vaginosis. Chin Med J (Engl).

[65] Teixeira GS, Carvalho FP, Arantes RM, Nunes AC, Moreira JL, Mendonca M, Almeida RB, Farias LM, Carvalho MA, Nicoli JR (2012). Characteristics of Lactobacillus and Gardnerella vaginalis from women with or without bacterial vaginosis and their relationships in gnotobiotic mice. J Med Microbiol.

[66] Castellarin M, Warren RL, Freeman JD, Dreolini L, Krzywinski M, Strauss J, Barnes R, Watson P, Allen-Vercoe E, Moore RA, Holt RA (2012). Fusobacterium nucleatum infection is prevalent in human colorectal carcinoma. Genome Res.

[67] Kostic AD, Gevers D, Pedamallu CS, Michaud M, Duke F, Earl AM, Ojesina AI, Jung J, Bass AJ, Tabernero J, Baselga J, Liu C, Shivdasani RA (2012). Genomic analysis identifies association of Fusobacterium with colorectal carcinoma. Genome Res.

[68] Tahara T, Yamamoto E, Suzuki H, Maruyama R, Chung W, Garriga J, Jelinek J, Yamano HO, Sugai T, An B, Shureiqi I, Toyota M, Kondo Y (2014). Fusobacterium in colonic flora and molecular features of colorectal carcinoma. Cancer Res.

[69] Rubinstein MR, Wang X, Liu W, Hao Y, Cai G, Han YW (2013). Fusobacterium nucleatum promotes colorectal carcinogenesis by modulating E-cadherin/beta-catenin signaling via its FadA adhesin. Cell Host Microbe.

[70] Keku TO, McCoy AN, Azcarate-Peril AM (2013). Fusobacterium spp. and colorectal cancer: cause or consequence?. Trends Microbiol.

[71] Kostic AD, Chun E, Robertson L, Glickman JN, Gallini CA, Michaud M, Clancy TE, Chung DC, Lochhead P, Hold GL, El-Omar EM, Brenner D, Fuchs CS (2013). Fusobacterium nucleatum potentiates intestinal tumorigenesis and modulates the tumor-immune microenvironment. Cell Host Microbe.

[72] Bashir A, Miskeen AY, Bhat A, Fazili KM, Ganai BA (2015). Fusobacterium nucleatum: an emerging bug in colorectal tumorigenesis. Eur J Cancer Prev.

[73] Zarco MF, Vess TJ, Ginsburg GS (2012). The oral microbiome in health and disease and the potential impact on personalized dental medicine. Oral Dis.

[74] Whitmore SE, Lamont RJ (2014). Oral bacteria and cancer. PLoS Pathog.

[75] Han YW (2015). Fusobacterium nucleatum: a commensal-turned pathogen. Curr Opin Microbiol.

[76] Gholizadeh P, Eslami H, Yousefi M, Asgharzadeh M, Aghazadeh M, Kafil HS (2016). Role of oral microbiome on oral cancers, a review. Biomed Pharmacother.

[77] Nagy KN, Sonkodi I, Szoke I, Nagy E, Newman HN (1998). The microflora associated with human oral carcinomas. Oral Oncol.

[78] Groeger S, Domann E, Gonzales JR, Chakraborty T, Meyle J (2011). B7-H1 and B7-DC receptors of oral squamous carcinoma cells are upregulated by Porphyromonas gingivalis. Immunobiology.

[79] Binder Gallimidi A, Fischman S, Revach B, Bulvik R, Maliutina A, Rubinstein AM, Nussbaum G, Elkin M (2015). Periodontal pathogens Porphyromonas gingivalis and Fusobacterium nucleatum promote tumor progression in an oral-specific chemical carcinogenesis model. Oncotarget.

[80] Takahashi N (2015). Oral Microbiome Metabolism: From "Who Are They?" to "What Are They Doing?". J Dent Res.

[81] Kolenbrander PE (2000). Oral microbial communities: biofilms, interactions, and genetic systems. Annu Rev Microbiol.

[82] Ximenez-Fyvie LA, Haffajee AD, Socransky SS (2000). Microbial composition of supra- and subgingival plaque in subjects with adult periodontitis. J Clin Periodontol.

[83] Washio J, Ogawa T, Suzuki K, Tsukiboshi Y, Watanabe M, Takahashi N (2016). Amino acid composition and amino acid-metabolic network in supragingival plaque. Biomed Res.

[84] Washio J, Takahashi N (2016). Metabolomic Studies of Oral Biofilm, Oral Cancer, and Beyond. Int J Mol Sci.

[85] Tian L, Sato T, Niwa K, Kawase M, Mayanagi G, Washio J, Takahashi N (2016). PCR-dipstick DNA chromatography for profiling of a subgroup of caries-associated bacterial species in plaque from healthy coronal surfaces and periodontal pockets. Biomed Res.

[86] Wang Q, Gao P, Wang X, Duan Y (2014). The early diagnosis and monitoring of squamous cell carcinoma via saliva metabolomics. Sci Rep.

[87] Jorth P, Turner KH, Gumus P, Nizam N, Buduneli N, Whiteley M (2014). Metatranscriptomics of the human oral microbiome during health and disease. MBio.

[88] Vital M, Howe AC, Tiedje JM (2014). Revealing the bacterial butyrate synthesis pathways by analyzing (meta)genomic data. MBio.

[89] Ang MY, Dutta A, Wee WY, Dymock D, Paterson IC, Choo SW (2016). Comparative Genome Analysis of Fusobacterium nucleatum. Genome Biol Evol.

[90] Sivan A, Corrales L, Hubert N, Williams JB, Aquino-Michaels K, Earley ZM, Benyamin FW, Lei YM, Jabri B, Alegre ML, Chang EB, Gajewski TF (2015). Commensal Bifidobacterium promotes antitumor immunity and facilitates anti-PD-L1 efficacy. Science.

[91] Ferris RL, Blumenschein G, Fayette J, Guigay J, Colevas AD, Licitra L, Harrington K, Kasper S, Vokes EE, Even C, Worden F, Saba NF, Iglesias Docampo LC (2016). Nivolumab for Recurrent Squamous-Cell Carcinoma of the Head and Neck. N Engl J Med.

[92] Human Microbiome Project Consortium (2012). A framework for human microbiome research. Nature.

[93] Hoque MO, Lee CC, Cairns P, Schoenberg M, Sidransky D (2003). Genome-wide genetic characterization of bladder cancer: a comparison of high-density single-nucleotide polymorphism arrays and PCR-based microsatellite analysis. Cancer Res.

[94] Daquigan N

[95] Pfefer T

[96] Ottesen A, Ramachandran P, Reed E, White JR, Hasan N, Subramanian P, Ryan G, Jarvis K, Grim C, Daquiqan N, Hanes D, Allard M, Colwell R (2016). Enrichment dynamics of Listeria monocytogenes and the associated microbiome from naturally contaminated ice cream linked to a listeriosis outbreak. BMC Microbiol.

[97] Navas-Molina JA, Peralta-Sanchez JM, Gonzalez A, McMurdie PJ, Vazquez-Baeza Y, Xu Z, Ursell LK, Lauber C, Zhou H, Song SJ, Huntley J, Ackermann GL, Berg-Lyons D (2013). Advancing our understanding of the human microbiome using QIIME. Methods Enzymol.

[98] Shannon P, Markiel A, Ozier O, Baliga NS, Wang JT, Ramage D, Amin N, Schwikowski B, Ideker T (2003). Cytoscape: a software environment for integrated models of biomolecular interaction networks. Genome Res.

[99] Boutaga K, van Winkelhoff AJ, Vandenbroucke-Grauls CM, Savelkoul PH (2005). Periodontal pathogens: a quantitative comparison of anaerobic culture and real-time PCR. FEMS Immunol Med Microbiol.

[100] Oksanen J, Blanchet G, Friendly M, Kindt R, Legendre P, McGlinn D, Minchin PR, O’Hara RB, Simpson GL, Solymos P, Stevens MHH, Szoecs E, Wagner H https://CRANR-projectorg/package=vegan.

[101] McMurdie PJ, Holmes S (2013). phyloseq: an R package for reproducible interactive analysis and graphics of microbiome census data. PLoS One.

