# Supplementary material for: High-resolution microbiome profiling uncovers Fusobacterium nucleatum, Lactobacillus gasseri/johnsonii, and Lactobacillus vaginalis associated to oral and oropharyngeal cancer in saliva from HPV positive and HPV negative patients treated with surgery and chemo-radiation
Source: Oncotarget. 2017 Sep 7;8(67):110931–48. doi: 10.18632/oncotarget.20677 (PMC5762296; doi:10.18632/oncotarget.20677)
Supplement: Supplementary file 1 [file oncotarget-08-110931-s001.pdf]

High-resolution microbiome profiling uncovers *Fusobacterium nucleatum*, *Lactobacillus gasseri/johnsonii*, and *Lactobacillus vaginalis* associated to oral and oropharyngeal cancer in saliva from HPV positive and HPV negative patients treated with surgery and chemo-radiation

SUPPLEMENTARY MATERIALS

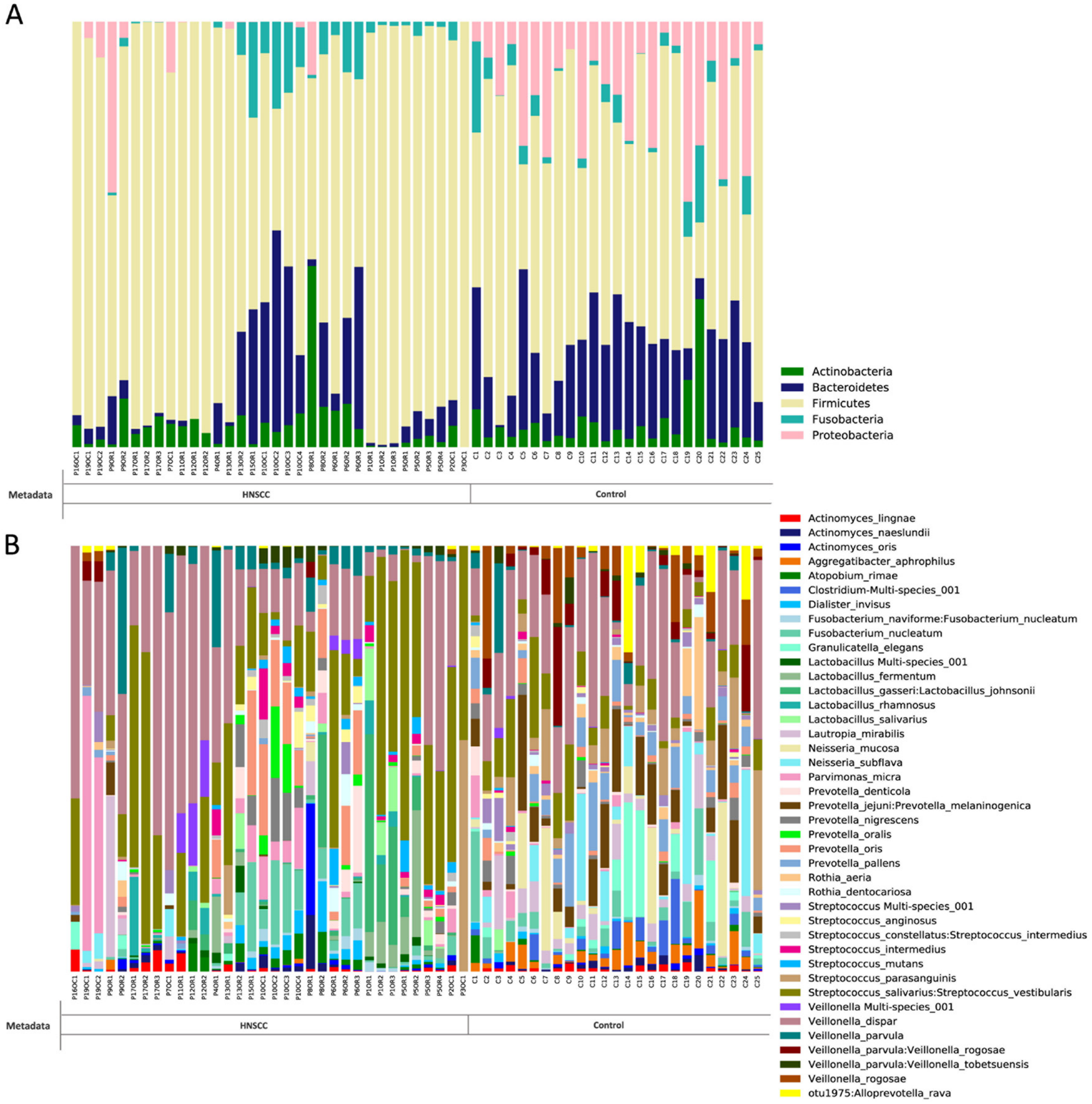

Supplementary Figure 1: (A-B) Taxonomic profiles at the phyla (A) and (B) species levels identified with high resolution profiling of 16S rRNA Next Generation Sequencing data in saliva from HNSCC and control samples.

(*Continued*)

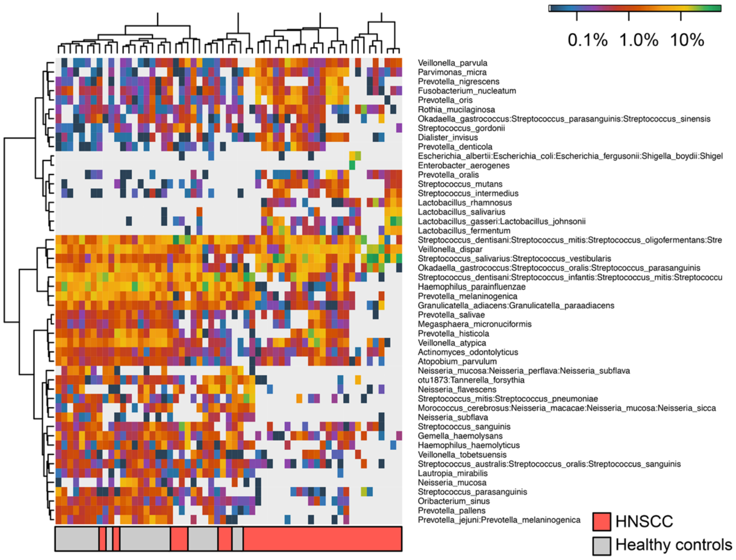

Supplementary Figure 1 (*Continued*): (C) Hierarchical clustering of taxonomic profiles (top 50 species level calls) shows that the salivary microbiome is distinct in HNSCC, with significant enrichment of Lactobacillus spp, Parvimonas micra, Streptococcus mutans, and Fusobacterium nucleatum ($p < 0.05$) in HNSCC samples and overall reduced alpha diversity ($p < 1e-4$).

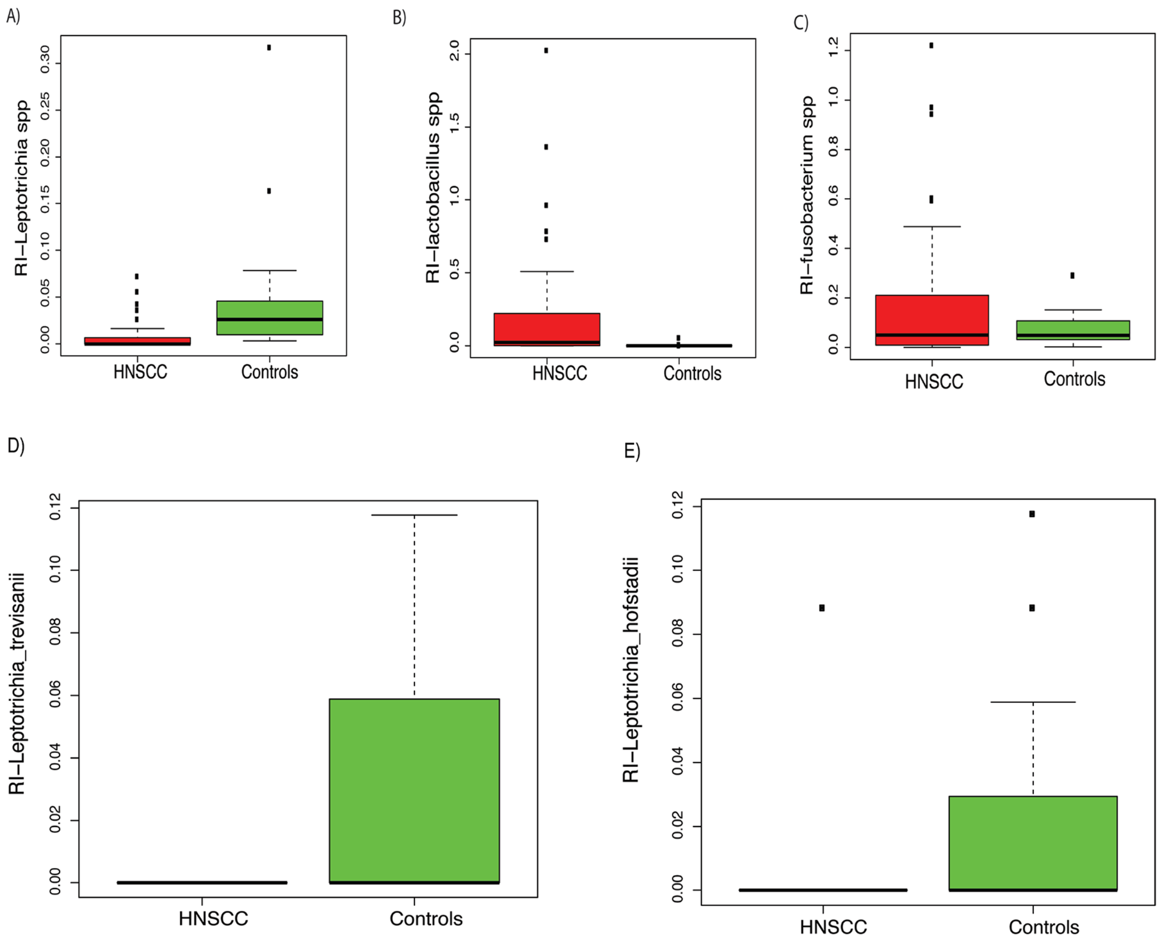

Supplementary Figure 2: Boxplots showing enrichment of several bacterial species in saliva from head and neck squamous cell carcinoma: **(A)** *Leptotrichia* spp; **(B)** *Lactobacillus* spp; **(C)** *Fusobacterium* spp; **(D)** *Leptotrichia trevisanii* and **(E)** *Leptotrichia hofstadii*. The boxplots support two novel findings: 1) A larger percentage of *Fusobacterium_nucleatum* is seen together with a highly significant depletion of *Leptotrichia trevisanii* and *Leptotrichia hofstadii* in HNSCC compared to controls in the Hopkins cohort.

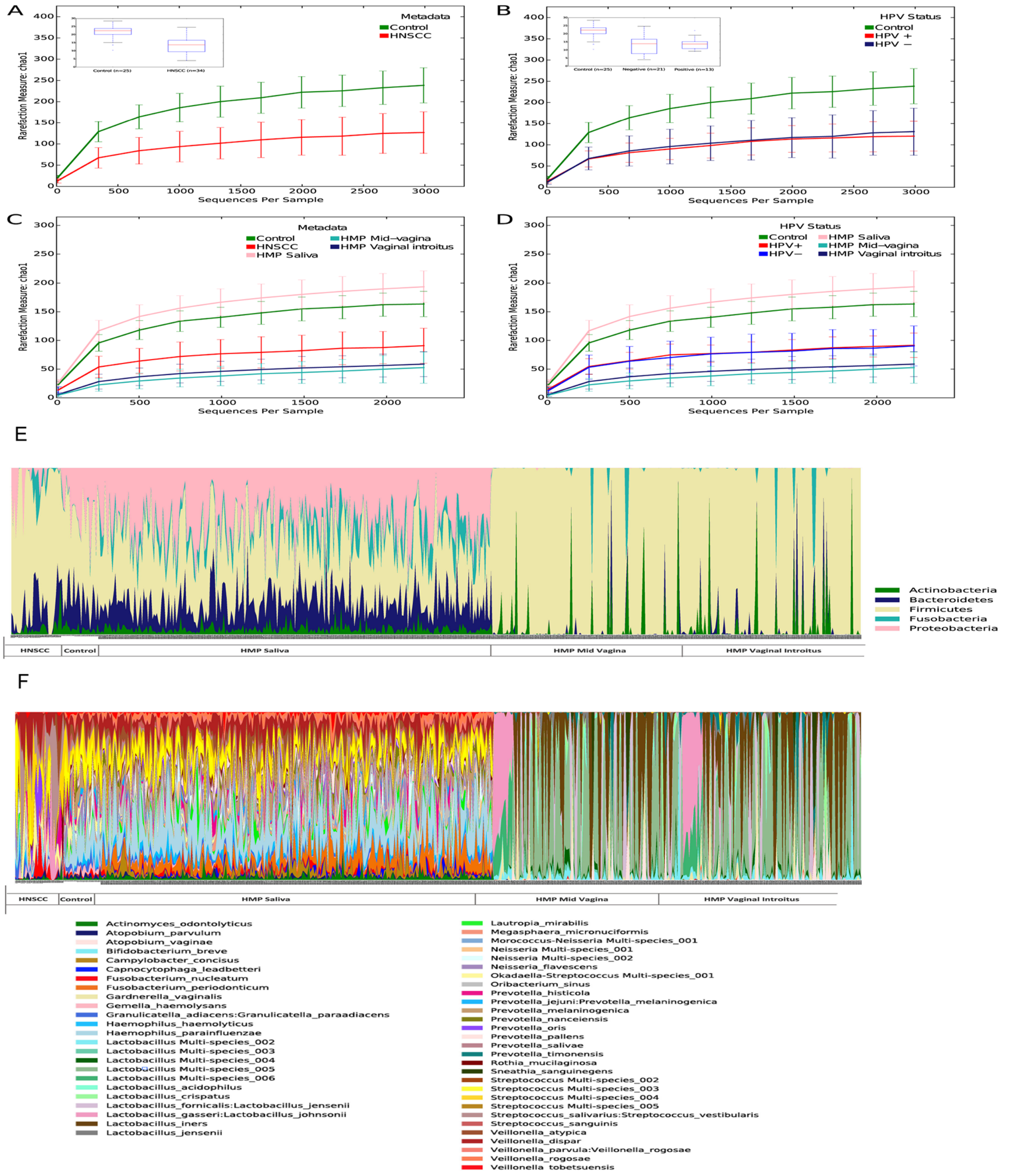

Supplementary Figure 3: Chao1 richness estimator rarefaction curves of species richness **(A-D)** and area plots of taxonomic summary for phyla **(E)** and species-level **(F)** showing microbial differences in saliva from Controls and head and neck squamous cell carcinoma patients (HNSCC). Microbial communities from JHU Control samples display significantly higher alpha richness ($p<0.001$) than HNSCC samples (A); There are no differences in species richness when comparing HPV positive and HPV negative samples (B); Saliva from normal HMP participants and JHU Controls had higher species richness than HNSCC and HMP vaginal samples (C); No significant differences were observed ($p=0.15$) when comparing HPV positive and HPV negative HNSCC samples with JHU Control and HMP saliva and vaginal samples (D). There were no differences in the microbial phyla present in the saliva control samples from JHU and saliva from participants in the HMP observed in the area plots of taxonomic summary for phyla (E). At the species level there were novel differences observed in the saliva samples from HNSCC patients with tumors from different anatomic sites. Bacteria commonly seen in the vaginal flora were found in saliva samples from a subset of Oropharyngeal Squamous Cell Carcinoma (OPSCC) patients (F).

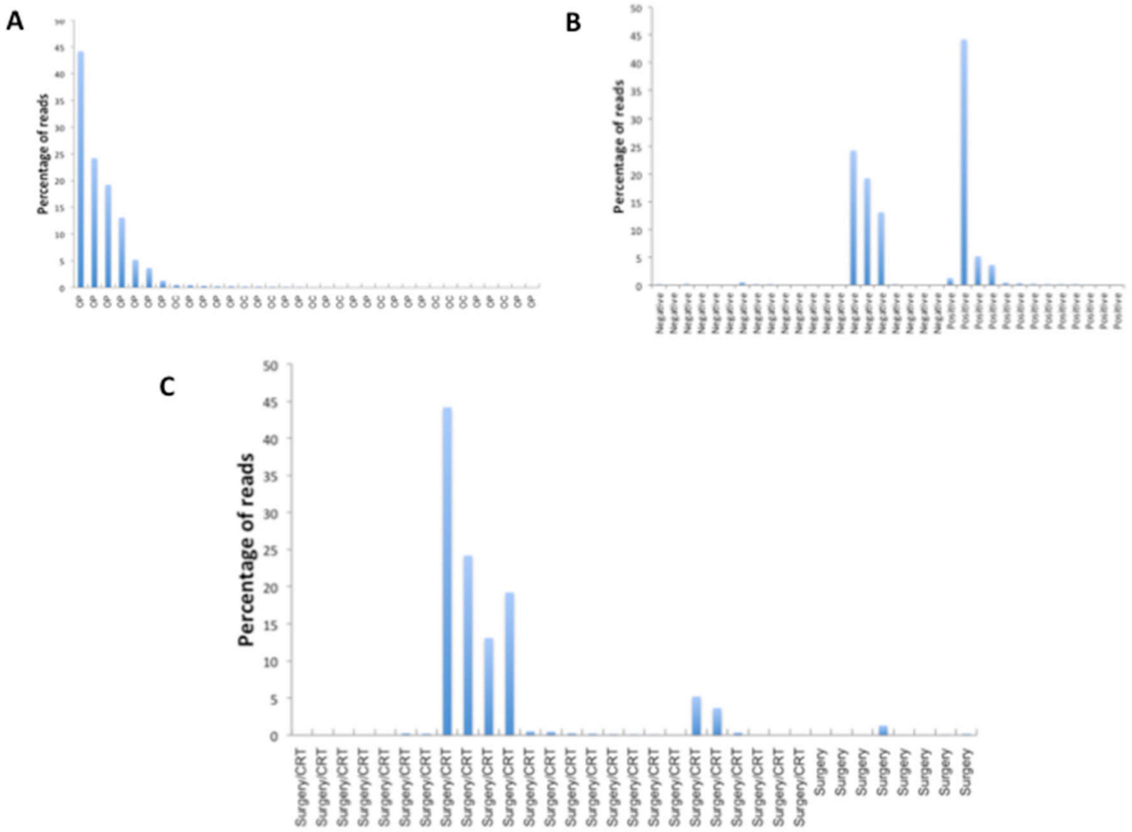

Supplementary Figure 4: (A-C) Percentage of significant Lactobacillus_gasseri:Lactobacillus_johnsonii 16S rRNA Next Generation Sequencing reads in saliva from Head and Neck Squamous Cell Carcinoma patients by anatomic site (A); by HPV status (B); and by Type of Treatment (C).

A

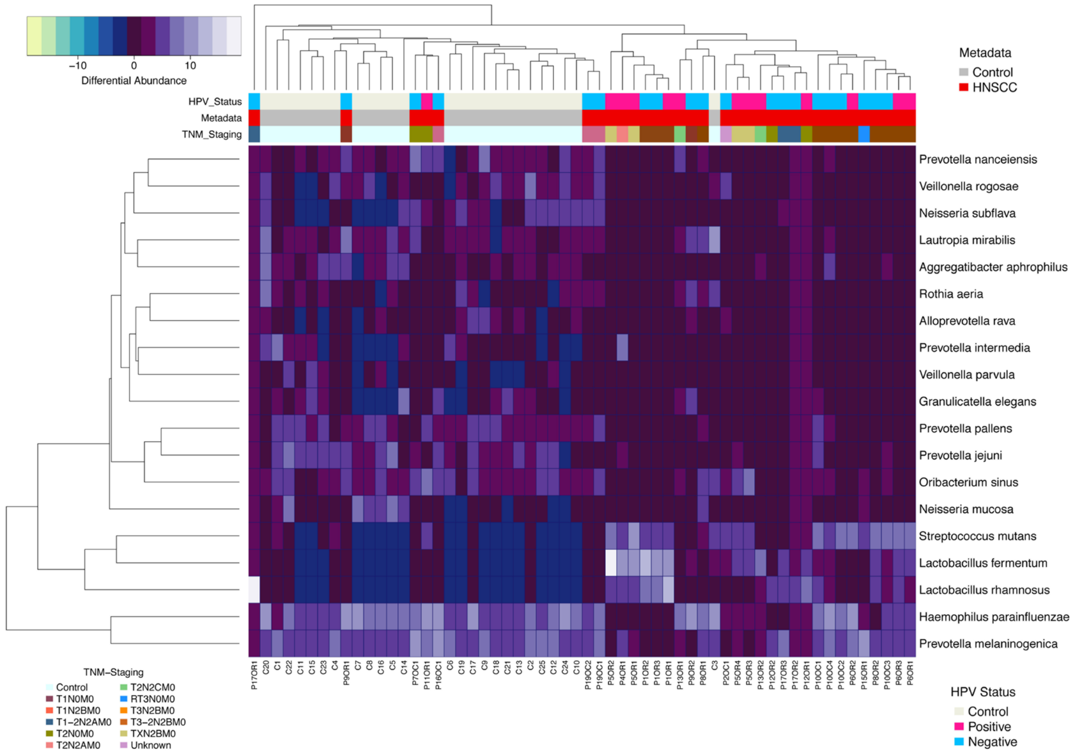

B

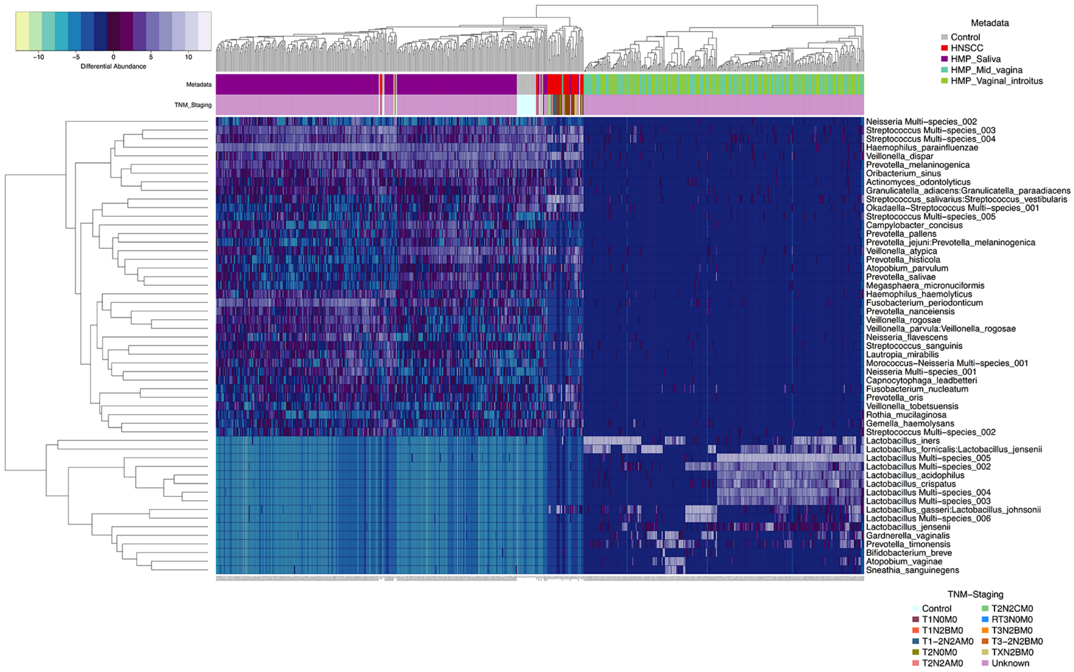

Supplementary Figure 5: (A) Single species heatmap using Euclidean distance to cluster Centralized log ratios (CLRs) of 19 statistically significant OTUs with the Hierarchical Unweighted Pair Group Method with Arithmetic Mean (UPGMA), shows that Streptococcus mutants, Lactobacillus fermentum and Lactobacillus rhamnosus are enriched in HNSCC samples. **(B)** A single species and multiple ambiguous Resphera assignment heatmap, using Euclidean distance to cluster Centralized Log Ratios of 53 statistically significant OTUs out of the 573 OTUs for JHU control and HNSCC samples' OTUs ($p<0.0001$) with the Hierarchical Unweighted Pair Group Method with Arithmetic Mean (UPGMA), shows that Streptococcus_salivarius: Streptococcus_vestibularis, Fusobacterium nucleatum, Prevotella oris, Rothia_mucilaginosa, and Lactobacilus_gasseri:Lactobacillus_johnsonni are enriched in HNSCC samples, when compared to normal saliva samples from JHU and HMP. We also observed a loss of enrichment of Prevotella_jejuni:Prevotella melaninogenica, and Prevotella_ pallens in HNSCC.

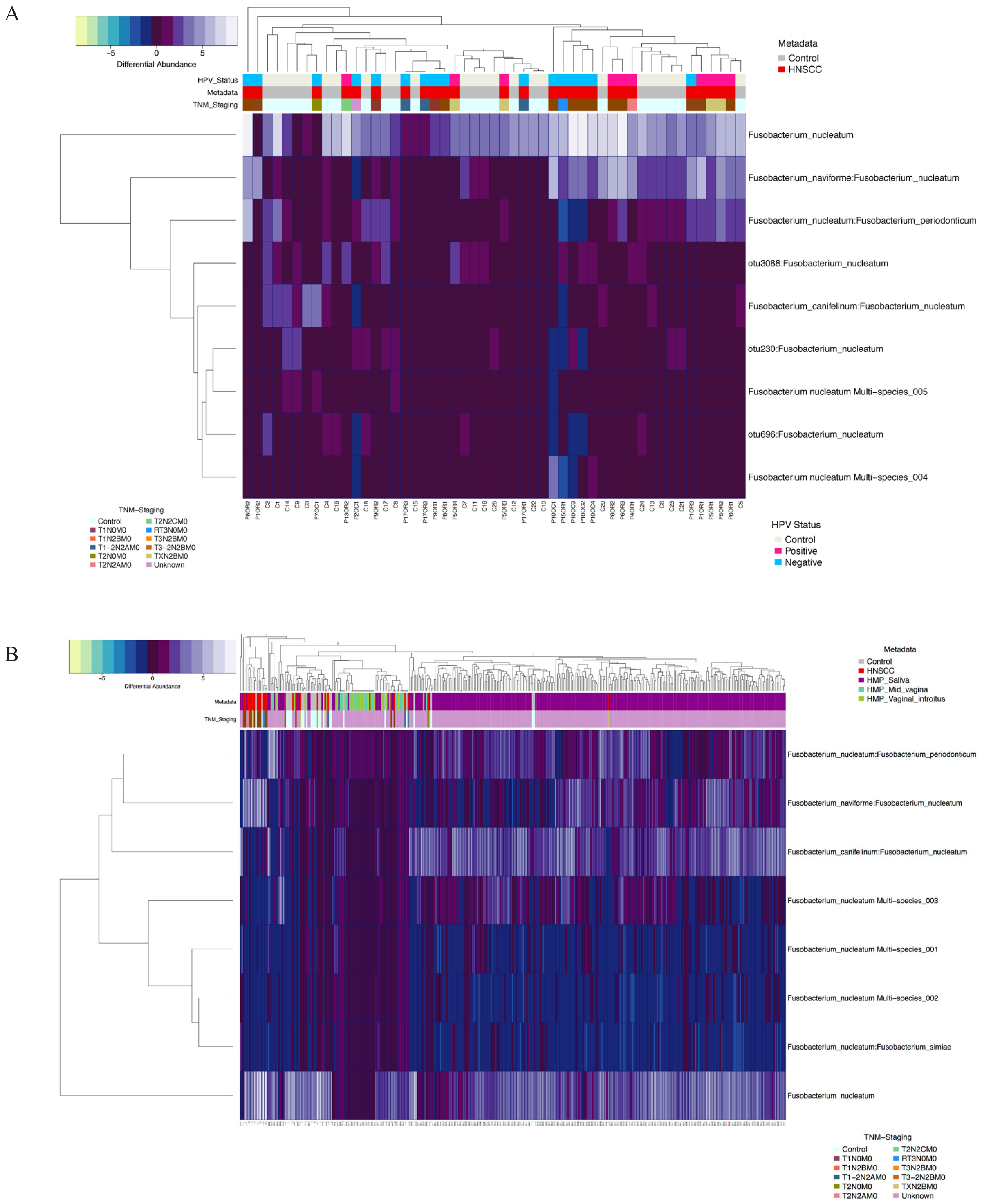

Supplementary Figure 6: (A) Heatmap differential abundance of significant (p<0. 0001) Fusobacterium species' OTUs in HNSCC when compared to JHU control samples with the variance stabilization method of QIIME's 1.9.1 and DESeq2 normalization for data after logarithmic transformation, shows enrichment of enrichment of Fusobacterium nucleatum and Fusobacterium naviforme in a subset of HNSCC samples. Fusobacterium species, such as F. canifelium, F. nucleatum and F. naviforme were differentially abundant across all samples. **(B)** Heatmap differential abundance of significant (p<0.0001) Fusobacterium species' OTUs in HNSCC when compared to JHU control samples and saliva, mid_vagina and vaginal_introitus samples from normal HMP participants with the variance stabilization method of QIIME's 1.9.1 and DESeq2 normalization for data after logarithmic transformation, shows differential enrichment of Fusobacterium nucleatum and Fusobacterium naviforme in 34 HNSCC samples when compared to 25 normal JHU saliva samples, as well as 290 saliva samples and 249 vaginal samples from the HMP.

(*Continued*)

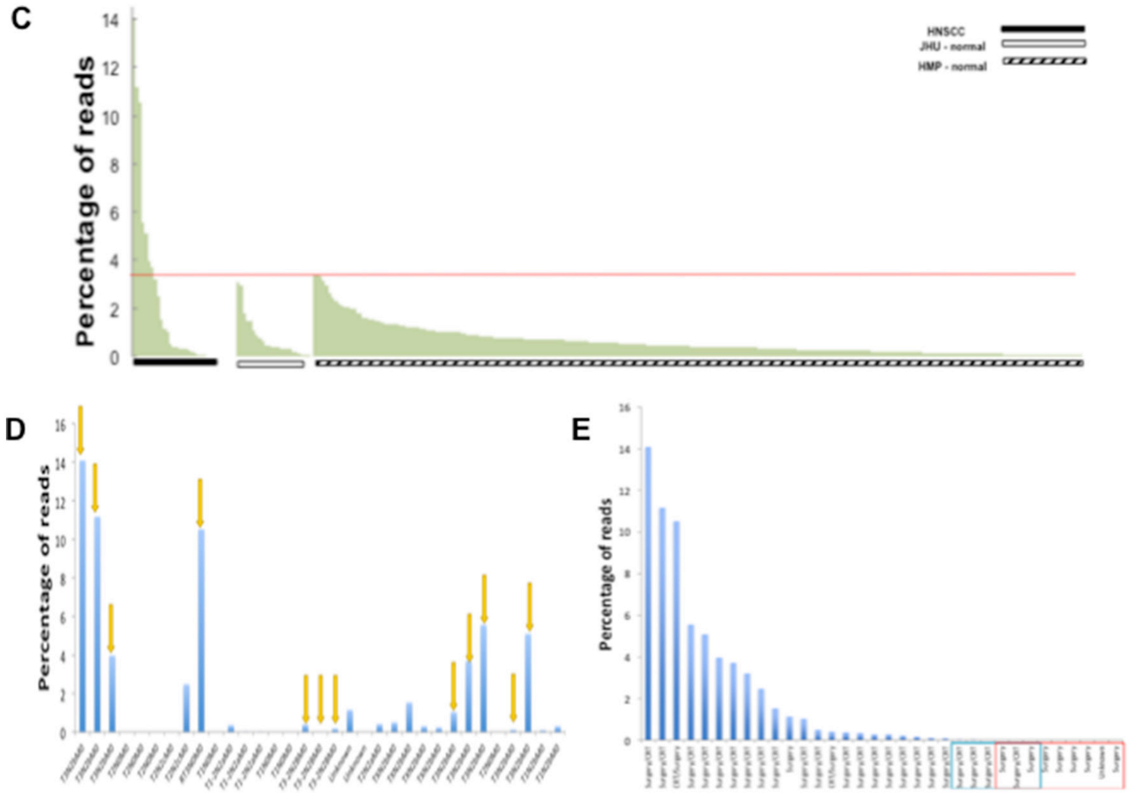

Supplementary Figure 6 (*Continued*): (C) The relative enrichment of Fusobacterium_nucleatum in HNSCC saliva when compared to saliva controls from Hopkins and from HMP; **(D)** by tumor size and nodal involvement according to TNM staging; **(E)** and in patients treated with surgery and chemo-radio-therapy when compared with patients only treated with surgical removal of the tumor.

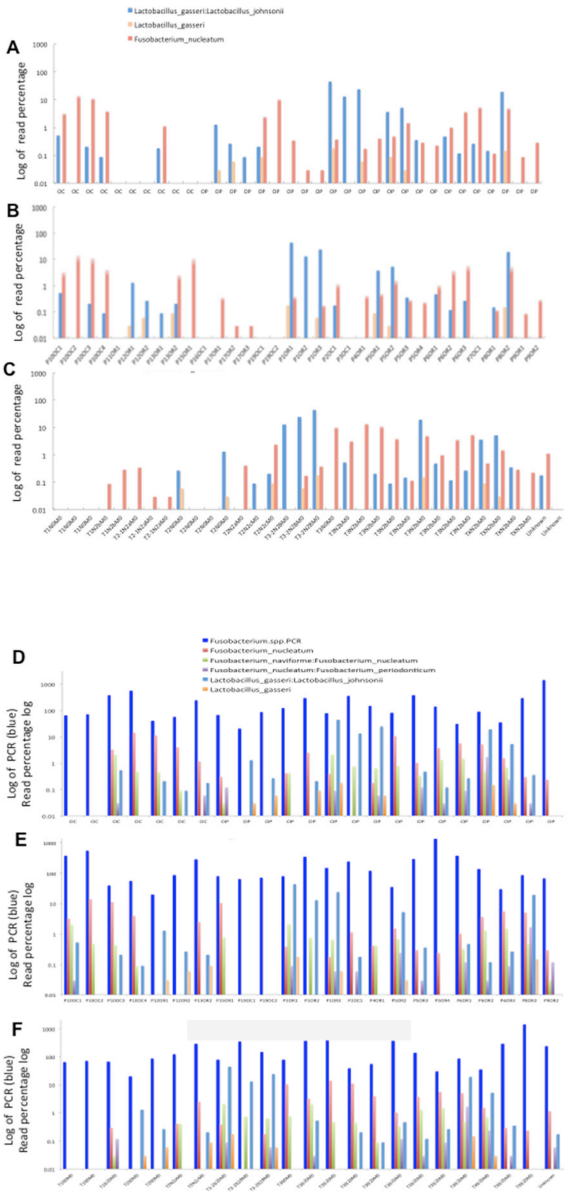

Supplementary Figure 7: (A-C) Relative enrichment of significant OTUs 16S rRNA NGS reads of Fusobacterium_nucleatum, Lactobacillus_gasseri:Lactobacillus_johnsonni, and Lactobacillus gasseri, in HNSCC saliva by (A) anatomic site, (B) longitudinal sampling, and (C) TNM stage. Units on the Y-axis are the logarithm of the percentage of significant reads. **(D-F)** Relative enrichment of Fusobacterium_spp, quantified by PCR amplification, juxtaposed to percentage of significant OTUs 16S rRNA NGS reads of Fusobacterium_nucleatum, Fusobacterium_naviforme:Fusobacterium_nucleatum, Fusobacterium_nucleatum:Fusobacterium_periodontecum, Lactobacillus_gasseri:Lactobacillus_johnsonni, and Lactobacillus gasseri, in HNSCC saliva by (D) anatomic site, (E) longitudinal sampling, and (F) TNM stage. Units on the Y-axis are the logarithm of the percentage of significant reads or the logarithm of relative PCR quantification for Fusobacterium_spp (blue).

Supplementary Table 1a: Number of Johns Hopkins Hospital patients, number of saliva samples, total number of sequences and total number of significant Operational Taxonomic Units for all Resphera-assigned raw counts at the single species level, by histology type and HPV status

Sample type (Histology+HPV status)	Number of Patients	Number of Samples	Total Number of sequences	Total Number of OTUs
Normal Mucosa (Control) HPV Negative	25	25	31,196	67
Oropharynx Squamous cell carcinoma (OPSCC) HPV Negative	4	11	7,192	47
Oropharynx Squamous cell carcinoma (OPSCC) HPV Positive	7	13	7,278	41
Oral Cavity Squamous cell carcinoma (OSCC) HPV Negative	6	10	4,828	55
**Grand Total**	42	59	50,494	210

Supplementary Table 1b: Number of Human Microbiome Project participants, number of samples, total number of sequences and Operational Taxonomic Units (OTUs) for all Resphera-assigned raw counts at the single species level, by body compartment

Body compartment	Number of Participants	Number of Samples	Total Number of sequences	Total Number of OTUs
Saliva	154	265**	252,764	67
Mid-Vagina	79*	128**	17,654	35
Vaginal Introitus	73*	121**	17,989	31
**Grand Total**	154	514	288,407	133

*Subset of 154 participants.
** Longitudinal samples.

Supplementary Table 2: Head and neck cancer patients characteristics

See Supplementary File 1

Supplementary Table 3: Patient characteristics for repeated samples analysis

See Supplementary File 2
